# A Machine Learning Model to Predict Post-Operative Intensive Care Unit Admission in Patients with Cancer Based on Clinical Characteristics and Hematologic Parameters Data

**DOI:** 10.3390/jcm15082898

**Published:** 2026-04-10

**Authors:** Jiaxin Cao, Zengfei Xia, Qun Chen, Chaozhuo Lin, Ting Yang, Fan Luo

**Affiliations:** 1 Department of Anesthesiology, State Key Laboratory of Oncology in South China, Guangdong Key Laboratory of Nasopharyngeal Carcinoma Diagnosis and Therapy, Guangdong Provincial Clinical Research Center for Cancer, Sun Yat-sen University Cancer Center, Guangzhou 510060, China; caojx@sysucc.org.cn; 2 Department of Experimental Research, State Key Laboratory of Oncology in South China, Guangdong Key Laboratory of Nasopharyngeal Carcinoma Diagnosis and Therapy, Guangdong Provincial Clinical Research Center for Cancer, Sun Yat-sen University Cancer Center, Guangzhou 510060, China; xiazf@sysucc.org.cn; 3 Department of Clinical Research, State Key Laboratory of Oncology in South China, Guangdong Key Laboratory of Nasopharyngeal Carcinoma Diagnosis and Therapy, Guangdong Provincial Clinical Research Center for Cancer, Sun Yat-sen University Cancer Center, Guangzhou 510060, China; chenqun@sysucc.org.cn (Q.C.); lincz@sysucc.org.cn (C.L.); yangting@sysucc.org.cn (T.Y.); 4 Department of Intensive Care Unit, State Key Laboratory of Oncology in South China, Guangdong Key Laboratory of Nasopharyngeal Carcinoma Diagnosis and Therapy, Guangdong Provincial Clinical Research Center for Cancer, Sun Yat-sen University Cancer Center, Guangzhou 510060, China

**Keywords:** cancer, intensive care unit, machine learning model, catboost

## Abstract

**Background and Objectives:** The prioritization of intensive care unit (ICU) admission following surgery for cancer is controversial. There is an urgent need to develop an appropriate clinical predictive model to aid in making ICU admission decisions after surgery. **Materials and Methods:** Four model strategies were used to build post-operative ICU admission predictive models: SVM, Catboost, ANN, and KNN. Internal verification was used for model evaluation at a ratio of 7:3. The area under the curve (AUC) value, calibration plots, and decision curve analysis were employed to assess the performance and clinical usefulness of the model. **Results:** The ICU group of patients with cancer who underwent surgery showed better prognosis for disease-free survival (DFS, *p* = 0.0008) and overall survival (OS, *p* < 0.0001). Cox multivariate analyses validated that lower baseline RBC, LDH, and CRP; higher baseline ALB, LCR, and lower post-operative LDH; higher post-operative HCT and ApoA-I; and higher fluctuating MCH independently predicted better DFS and OS (all *p* < 0.05). The AUC of the Catboost model was superior to that of the other models in the training cohort and internal validation cohort. Calibration plot and decision curve analysis indicated that the Catboost model possessed the best performance, with higher clinical utility, compared with other models. **Conclusions:** ICU admission after surgery was associated with superior survival in patients with cancer. The cost-effective Catboost model has promising clinical application but requires further clinical evaluation. Unravelling the cellular and molecular foundation of ICU admission might enable the development of more practical life-support strategies.

## 1. Introduction

Worldwide cancer incidence and mortality rates are on the rise [1]. Recent decades have seen gradual improvements in the survival of patients with early-stage cancer, mainly because of improvements in surgical techniques and advances in therapeutic strategies [2]. Despite careful pre-operative risk management and the use of modern surgical and anesthetic techniques, cancer surgery is still associated with poor survival, mostly related to specific comorbidities [3]. To extend the benefits of cancer surgery to a wider population, it is necessary to provide post-operative management strategies to reduce complications and improve survival.

Although intensive care (IC) can be lifesaving for certain patients, for other patients it is costly, non-beneficial, and even harmful [4]. According to the guidelines, admission to an intensive care unit (ICU) is recommended to be based only on the ability of a patient to benefit from such care [5]. Traditionally, after thoracic surgery, selective ICU admission can reduce complications and has a favorable impact on patient outcomes [3]. The prevalence of thoracic cancer has gradually increased in recent years; however, the ICU admission of post-operative patients with cancer remains a challenging issue. Importantly, the benefits these patients might derive from post-operative ICU must be taken into account.

The number of patients with cancer being admitted to an ICU is rising [6], with lung cancer accounting for 8% of all ICU admissions among patients with malignancies and 27% of those with solid cancer [7,8]. Currently, the benefit to cancer patients from ICU admission is a hot topic and is controversial. It is perceived that patients with cancer usually experience markedly worse ICU outcomes in comparison with patients with other diseases [9]. Moreover, among these patients, admission to the ICU for life-threatening events is widely believed to be of unlikely benefit, especially when ventilatory support is needed [10]. Nevertheless, Meert et al. showed that ICU admission was associated with meaningful survival in patients with cancer, and their prognosis improved substantially with time [3,11]. Another study reported that patients admitted to the ICU for post-operative care following major surgical resections generally have good outcomes with low hospital-based mortality [12]. Our investigation of these studies indicated that they primarily concentrated on the advantages of ICU admission for any-stage cancer patients with severe complications, yet they failed to address the benefits of ICU admission following surgery for early-stage cancer patients. Overall, clinical evidence regarding the advantages of ICU admission after cancer surgery remains scarce.

A more comprehensive understanding of how decisions are made regarding ICU admission after cancer surgery is required. Routinely acquired clinical data on post-operative outcomes could provide clinicians with better guidance to assess patients receiving oncological surgery and could aid the development of guidelines and policies. Leveraging a large population, this study aimed to explore associations between post-operative ICU admission and survival outcomes for patients with cancer, investigate the influences of clinical characteristics and hematological parameters on ICU admission, and further develop a model that predicts current institutional ICU triage patterns using routinely acquired clinical characteristics and hematological parameter data.

## 2. Materials and Methods

### 2.1. Design of the Study and Enrollment of Patients

**Figure 1:**
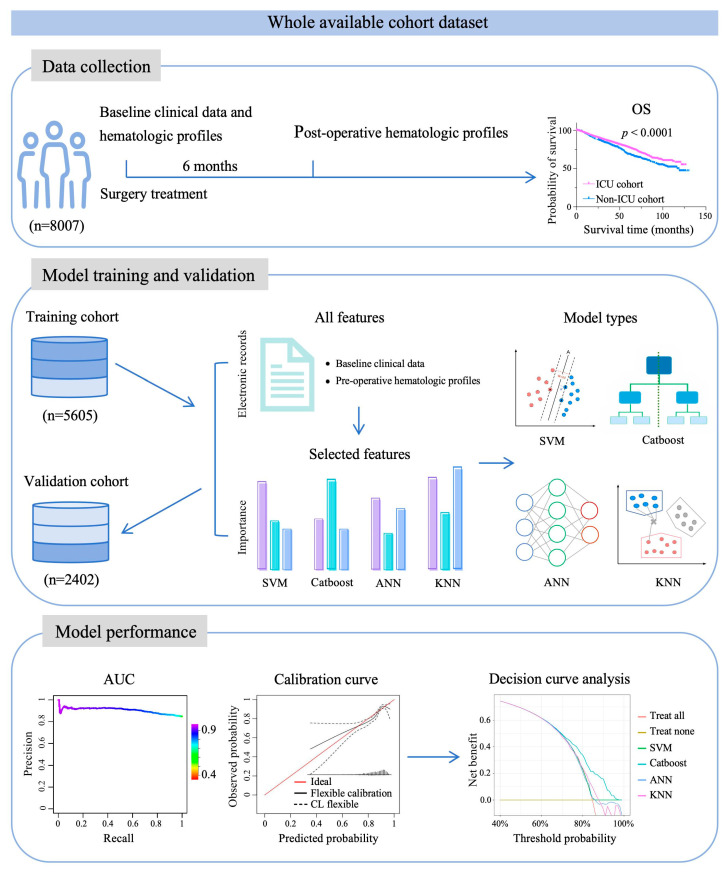
Study design for screening, training, and internal validation of the post-operative predictive ICU admission model.

In this study, we adopted a stepwise strategy with a screening, training, and internal validation design (Figure 1). A retrospective cohort of patients with cancer treated at the Sun Yat-sen University Cancer Center from January 2012 to December 2020 (n = 8007) was utilized. Patients who underwent surgery were randomly divided into the training dataset (for model development) and the model set (for internal validation) at a ratio of 7:3. We enrolled patients who received upfront curative-intent resection for cancer, who were aged ≥ 18 years or older. The exclusion criteria comprised the following: history of another malignancy (n = 124); baseline clinical characteristics (n = 1453) or hematological parameters (n = 2102) were not available; or follow-up data (n = 341) were missing. The investigation was carried out following the tenets of the Declaration of Helsinki and Good Clinical Practice Guidelines.

### 2.2. Clinical Data Collection

We collected clinical data: age, smoking status, sex, drinking status, hypertension, diabetes, family history, pathological type, surgical method, disease stage, adjuvant therapy, and ICU admission status (immediate post-anesthesia ICU transfer). ICU admission decisions at our center follow a standardized recommendation with selective non-compliance pattern that creates a quasi-experimental situation particularly valuable for our research question. Our main research focus is on thoracic surgeries; the departmental policy is that all patients undergoing major lung resection should be admitted to the ICU post-operatively. This policy was established due to high incidence of post-operative complications and rapid deterioration risk. Despite the standardized recommendation, some patients also did not enter the ICU. This variation arises from non-clinical factors that effectively create randomization-like conditions: patient/family refusal and ICU bed unavailability. Following surgery, follow-up of the patients was carried out regularly, comprising clinical assessment and imaging every 3 to 6 months. Disease-free survival (DFS) was defined as the period between surgical resection and verified radiological recurrence or death from the tumor. Overall survival (OS) was measured from the date of inclusion until the date of last follow-up or death. December 2023 was the date of the last patient follow-up. During the data collection process, data integrity was strictly controlled. For samples with missing indicator data, the complete-case analysis method was used for exclusion to reduce the interference of missing data on the model results.

### 2.3. Measuring Hematological Parameters in Blood Samples

After fasting, the patient’s whole blood was collected in an ethylene diamine tetraacetic acid (EDTA) anticoagulant-treated tube and analyzed within 30 min of collection. We measured 27 hematological parameters: Red blood cell (RBC), white blood cell (WBC), neutrophil, platelet (PLT), lymphocyte, C-reactive protein (CRP), monocyte, immune-inflammation index (SII), the neutrophil-lymphocyte ratio (NLR), the platelet/lymphocyte ratio (PLR), the lymphocyte-to-C-reactive protein ratio (LCR), and the lymphocyte/monocyte ratio (LMR), hemoglobin (HGB), hematocrit (HCT), mean corpuscular volume (MCV), mean corpuscular hemoglobin (MCH), serum albumin (ALB), globulin (GLOB), the ALB-to-GLOB ratio (AGR), total protein (TP), cholesterol (CHO), high-density lipoprotein-cholesterol (HDL-C), low-density lipoprotein-cholesterol (LDL-C), triglycerides (TG), apolipoprotein A-1 (ApoA-I), an apolipoprotein B (Apo-B), and lactate dehydrogenase (LDH). A combination of two or three hematological inflammation parameters was used to define SII, NLR, PLR, LCR, and LMR: SII  =  neutrophil  ×  platelet/lymphocyte; NLR  =  neutrophil/lymphocyte; PLR  =  platelet/lymphocyte; LCR = lymphocyt/C-reactive protein; LMR  =  lymphocyte/monocyte. Each patient provided peripheral blood samples at two time points: (i) within 1 week before surgery (baseline); (ii) 6 months after surgery. Written informed consent was received from all participants.

### 2.4. The Cox Models for Clinical Data and Hematological Parameters

Cox proportional hazards regression models were used to analyze the association between clinical data, hematological parameters, and the study outcomes (e.g., overall survival time, disease progression, etc.). The specific implementation process is as follows: First, the proportional hazards assumption of the finally constructed Cox regression model was tested using Schoenfeld residuals to evaluate whether the model met this core premise. Second, to avoid model estimation bias caused by multicollinearity among the selected variables, variance inflation factors (VIFs) were used to diagnose collinearity of the selected variables. To further verify the reliability of the model results and reduce the risk of overfitting, possibly caused by high-dimensional and correlated hematological variables, LASSO-penalized Cox regression was used as a complementary modeling strategy. The results were compared with those of the Cox model constructed by the stepwise regression method. The predictors and association trends obtained by the two methods were consistent, which further confirmed the validity and robustness of the study conclusions.

### 2.5. Machine Learning

To construct an optimal clinical data-based machine learning model to identify patients who might currently prioritize from ICU management, we collected complete clinical data and the hematological laboratory data at baseline. Before the development of machine learning models, the patients were assigned randomly into a training cohort (n = 5605) and a validation cohort (n = 2402) with a ratio of 7:3. Data gap handling was performed using the deletion method; samples with missing indicator data were deleted. During preprocessing, for most models, which are sensitive to feature scales, we applied standardization (using scale) to all continuous numerical features. As a tree-based model, Catboost does not require feature scaling, so we retained the original feature scales, which is consistent with common practice.

During construction of the machine learning model, four machine learning methods were implemented on the basis of the training cohort, including support vector machines (SVMs) [13], artificial neural network (ANN) [14], categorical boosting (Catboost), and K-Nearest Neighbor (KNN) [15]. The four models were chosen for their unique advantages and distinguished algorithm types, and the construction of the four models was independent from the three sets of data. Before constructing the final machine learning model, we identified the most critical factors for inclusion in the model in the training set and excluded features that tended not to affect the performance of the machine learning algorithm. For the SVM and KNN models, the overall feature importance in the model was assessed using the explain function of the DALEX R package, and the importance was represented as one minus the area under the curve (AUC) loss after permutation. For the Catboost model, the importance of features was calculated using the inline function of the Catboost R package. For the ANN model, the importance of each feature was scored as permutation feature importance using the permimp R package. The details of feature selection for each model and the cutoff values are shown in the Supplementary Table S1. With selected features serving as the inputs, the four machine learning models were trained using the training cohort. Hyperparameters of all models were optimized via grid search followed by 10-fold cross-validation. The details of overfitting control for each model are as follows: For the ANN model, dropout layers (dropout rate = 0.25) and early stopping (patience = 20) were added to mitigate overfitting; the Adam optimizer (batch size = 300, learning rate = 0.001) was applied together with the deep learning library Keras 2.4. For the SVM model, a Gaussian kernel was used, and a grid search over the cost and gamma parameters was performed using the tune.svm function with bootstrap resampling to select a model with good generalization ability. For the Catboost model, 1000 iterations were used, with the Logloss function and a learning rate of 0.01; overfitting was effectively prevented by early stopping (stopping patience = 20) and built-in L2 regularization (λ = 3), subsample = 0.8, and max tree depth = 4. For the KNN model, the optimal number of neighbors was selected by setting a dropout rate of 0.3 and early stopping with patience = 20. Then, the predictive performances of each model were tested on the training cohort and a validation cohort, separately. In each set of data, the four models were compared with each other to select the model with the best predictive performance in the training cohort and validation cohort. The optimal model was chosen by considering the AUC value of the Precision–Recall (PR) Curve, the calibration intercept, the calibration slope, the AUC value (95% confidence interval (CI), 95% prediction interval (PI)), and the Brier Score. Especially, the calibration curve was constructed using the predicted values adjusted with Platt scaling. For the PR-curve value, the closer the value is to 1.00, the bigger the area under the PR curve is, indicating a higher predictive power of the model, with ranges from 0.5 to 1.0. To balance misclassification costs, for the ensemble models, we explored a probability threshold to define classification decisions. What is more, in the training of individual models, the class weighting was also incorporated. Based on the differential clinical costs of false negatives and false positives, systematic threshold optimization was conducted, too. Finally, the resampling technique SMOTE was performed to analyze the sensitivity. These measures ensure that the model evaluation is not solely based on discrimination but also on practical utility for ICU triage.

To assess model performance, several other metrics were applied as supplementary tools. A calibration slope value close to 1.00 indicated a better confidence level. The smaller the Brier Score, the better the model’s predictive accuracy. Finally, the four models developed using the training cohort and validation cohort in each set of data were further compared among the training and validation cohorts, respectively, in the most comprehensive decision curve analysis (DCA).

### 2.6. Statistical Analysis

X-tile (Version 3.6.1) was used to find the optimal cut-off values. Categorical data were compared utilizing the chi-squared test, and continuous data were compared utilizing the independent *t*-test. The univariate and multivariate analyses employed Cox proportional hazards regression models. The multivariate regression model included variables with *p*-values less than 0.15 according to the univariate analysis, and the variables with NA values in the multivariate Cox regression results were not shown in the forest plot (SPSS Version 25, IBM, Armonk, NY, USA). The median DFS and OS were calculated employing the Kaplan–Meier method, and the log-rank test was used to compare the curves (GraphPad Prism 8, GraphPad Inc., La Jolla, CA, USA). The AUC with its 95% CI was calculated to assess the model’s predictive performance. DCA was used to compare the net benefit of each model. The model development and data processing were operated within the free statistical software of R (v4.4.3). The following R packages were used in this study: caret for the KNN model, catboost for the Catboost model, keras for the ANN model, e1071 for the SVM model, DALEX for feature explanation, and boost for Bootstrap. All statistical tests were two-sided, and statistical significance was indicated by a *p*-value less than 0.05.

## 3. Results

### 3.1. Baseline Characteristics of the Patients

This study included 8007 patients: 6799 in the post-operative ICU admission cohort (hereafter referred to as the ICU cohort (or ICU group)), and 1208 in the non-ICU admission cohort (hereafter referred to as the non-ICU cohort (or non-ICU group)). Supplementary Table S2 shows the detailed baseline characteristics of the patients.

In the ICU cohort, the patients had a median age of 64 years, and 3957 (58.20%) of them were male. The majority of the patients did not smoke (59.26%), drink alcohol (79.41%), did not have hypertension (80.59%), and were non-diabetic (89.72%). Only 21.91% of patients had a reported family history of cancer. In addition, 5187 (76.52%) patients had adenocarcinoma, and 1130 (16.67%) had squamous carcinoma. In terms of the surgical method used, 2418 (35.56%) patients received wedge resection, 3007 (44.17%) received a lobectomy, and 1374 (20.27%) received a pneumonectomy. Regarding the stage of the disease, 3779 (55.58%) patients had stage I disease, 1484 (21.83%) had stage II disease, 1412 (20.77%) had stage III disease, and 124 (1.82%) had stage IV disease. Regarding the adjuvant therapy administered, 402 (5.91%) patients received radiotherapy, 274 (4.03%) patients received immunotherapy, and 1366 (20.09%) patients received chemotherapy.

The non-ICU cohort consisted of 671 (55.55%) men and 537 (44.45%) women, with a median age of 57 years. Among them, 464 (38.41%) were smokers, while most patients were non-drinkers (78.97%), did not have hypertension (82.78%), and were non-diabetic (91.31%). Just 286 (23.68%) of them had a reported family history of cancer. The majority of patients had adenocarcinoma (76.99%) and received lobectomy surgery (44.53%). Regarding disease stage, 630 (52.15%) had stage I disease, 265 (21.94%) had stage II disease, 290 (24.01%) had stage III disease, and 23 (1.9%) had stage IV disease. The adjuvant therapy administered to these patients was radiotherapy (71/5.88%), immunotherapy (58/4.80%), and chemotherapy (272/22.52%). Overall, the two cohorts showed no significant differences.

### 3.2. Prognosis Comparison Between the Post-Operative ICU Group and Non-ICU Group

**Figure 2:**
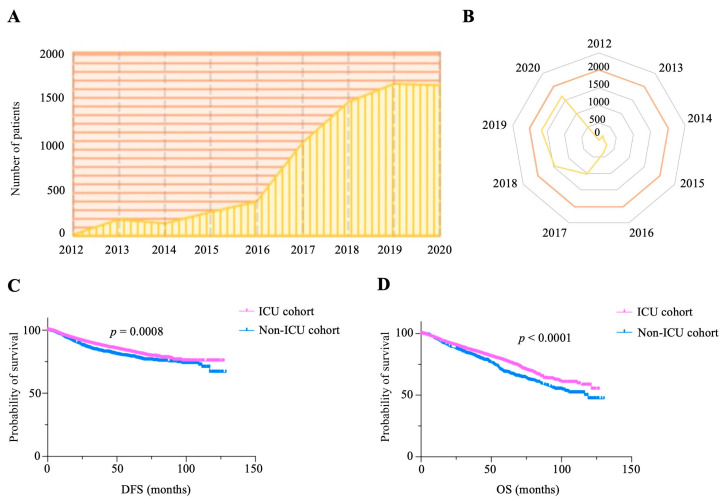
Kaplan–Meier survival curves for post-operative ICU and non-ICU groups. (**A**,**B**) Numbers of ICU admissions occupied by patients with cancer over time. (**C**) Significant difference in DFS between patients in the post-operative ICU and non-ICU groups (*p* = 0.0008). (**D**) Significant OS difference between patients in the post-operative ICU and non-ICU groups (*p* < 0.0001).

There was an increase in the proportion of patients with cancer admitted to the ICU after surgery from 2012 to 2020 (Figure 2A,B). The survival analysis found that ICU admission was associated with superior DFS prognosis for patients with cancer receiving surgery. The median DFS of patients in the ICU and non-ICU groups was undefined (*p* = 0.0008, Figure 2C). The DFS rates at 5 years were 82.637% and 78.861% for patients in the ICU and non-ICU groups, respectively. The DFS rates at 10 years were 75.390% and 66.500% for patients in the ICU and non-ICU groups, respectively. The median OS values of patients in the ICU and non-ICU groups were undefined and 116.4 months, respectively (*p* < 0.0001, Figure 2D). The OS rates at 5 years were 77.465% and 68.399% for patients in the ICU and non-ICU groups, respectively. The OS rates at 10 years were 57.867% and 46.883% for patients in the ICU and non-ICU groups, respectively.

### 3.3. Effects of Clinical Characteristics on DFS and OS in Patients Between the IC and Non-ICU Cohorts

**Figure 3:**
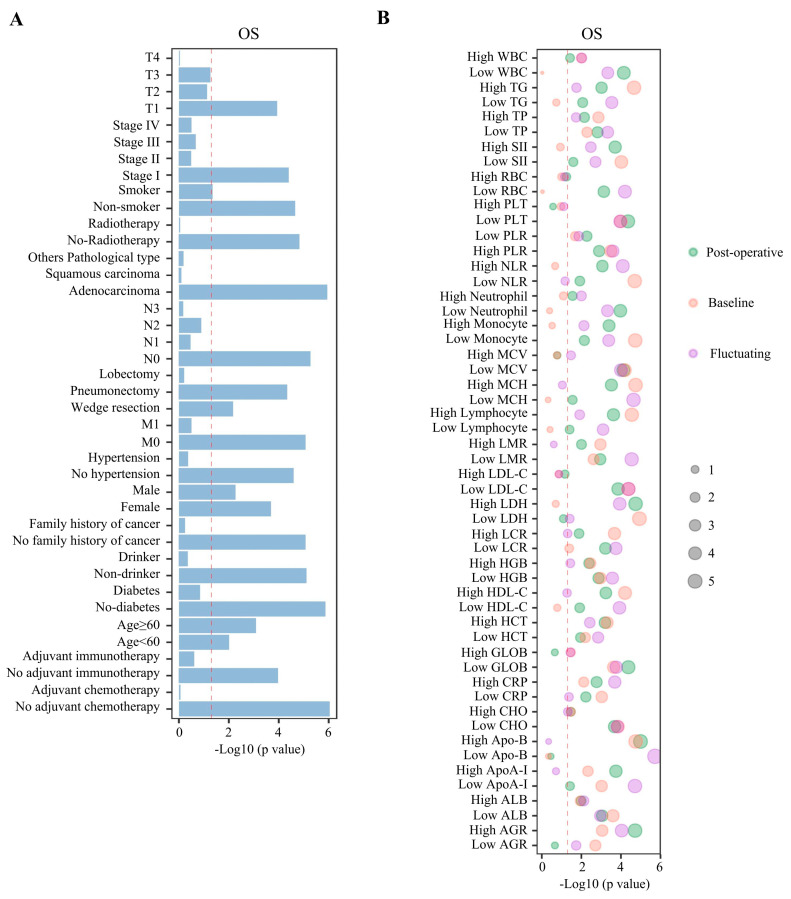
Effects of clinical characteristics and baseline/post-operative/ fluctuating hematological parameters on OS in patients between the ICU and non-ICU cohorts. (**A**) Subgroup analysis showing superior OS in the ICU group relative to that in the non-ICU group for the above clinical characteristics. (**B**) Subgroup analysis showing superior OS in the ICU group relative to that in the non-ICU group for the above hematological parameters. *p*-values were generated with the log-rank test.

Further subgroup analysis (Supplementary Table S3, Supplementary Figure S1A) showed that DFS was superior in the ICU group compared with that in the non-ICU group for patients who were older; female; non-smokers; non-drinkers; not hypertensive; non-diabetic; had no family history of cancer; had adenocarcinoma; were at T1, N0, M0, or stage I; and did not receive radiotherapy, adjuvant chemotherapy, or adjuvant immunotherapy with HR < 1; but in patients who received a pneumonectomy, DFS was poorer in the ICU group compared with that in the non-ICU group (HR = 1.42). Moreover, regardless of whether they were <60 years or ≥60 years; male or female; a non-smoker or a smoker; a non-drinker; non-hypertensive, non-diabetic; had no family history of cancer; had adenocarcinoma; received wedge resection or pneumonectomy; were at T1, N0, M0, or stage I; or did not receive radiotherapy, adjuvant chemotherapy, or adjuvant immunotherapy, patients in the ICU group were significantly associated with longer OS; but in patients who received a pneumonectomy, OS was poorer in the ICU group compared with that in the non-ICU group (HR = 1.09) (Supplementary Table S3, Figure 3A). For the other subgroups of clinical characteristics, there were no significant differences in terms of survival between ICU and non-ICU groups.

### 3.4. Peripheral Blood Hematological Parameters Comparisons Pre- and Post-Surgery in Patients with Cancer

**Figure 4:**
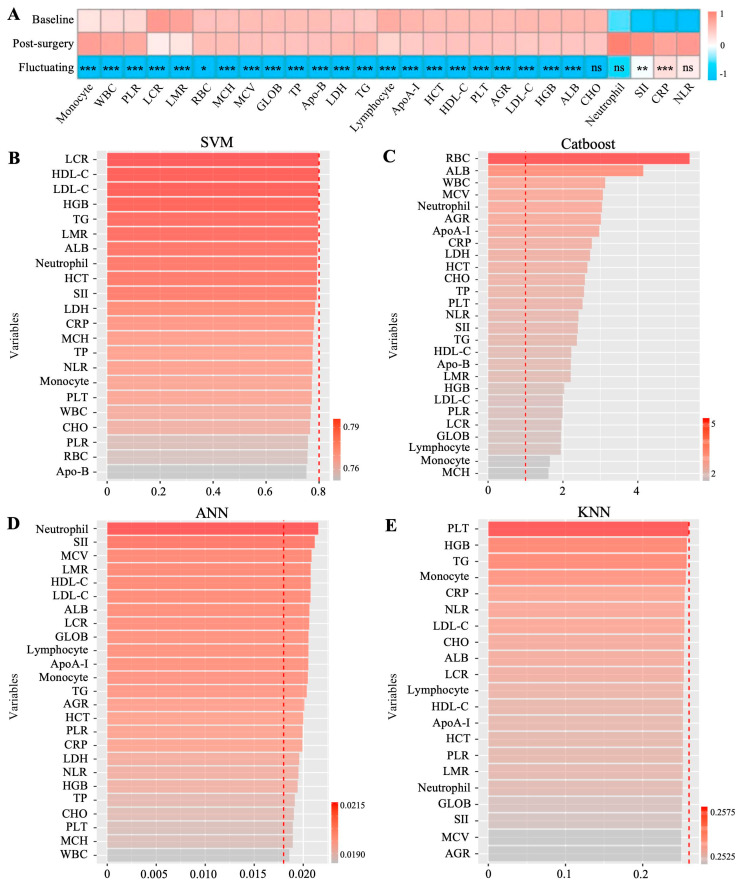
Screening for importance scores of hematological parameters used to predict ICU hospitalization modeling. (**A**) Significant fluctuation of post-operative hematological parameters in patients with cancer. (**B**–**E**) The importance of selected hematological parameters for SVM, Catboost, ANN, and KNN predictive models based on baseline hematological parameters. The importance of variables for Catboost and ANN was presented as importance value, for SVM and KNN as 1-AUC loss after permutation. * *p* < 0.05, ** *p* < 0.01, *** *p* < 0.001.

Compared with the baseline hematological parameters, we observed significant fluctuations of RBC, WBC, PLT, lymphocyte, CRP, monocyte, SII, PLR, LCR, LMR, HGB, HCT, MCV, MCH, ALB, GLOB, AGR, TP, HDL-C, LDL-C, TG, ApoA-I, Apo-B, and LDH (*p* < 0.05) in patients with cancer post-surgery. Only neutrophil, NLR, and CHO levels showed no significant differences pre- and post-surgery (Figure 4A, Supplementary Table S4). The mean pre-surgery and post-surgery levels of hematological parameters are shown in Supplementary Table S4.

### 3.5. The Impact of Hematological Parameters and Their Dynamic Changes on DFS and OS in Patients Between the ICU and Non-ICU Cohorts

X-tile was used to determine the cut-offs for the hematological parameter levels before surgery, allowing them to be classified as ‘high’ or ‘low’ (Supplementary Table S5). We then analyzed the associations between the baseline, post-operative, and dynamic changes in the levels of the hematological parameters and prognosis in the ICU and non-ICU groups (Supplementary Tables S6–S8). For the baseline hematological parameter data, it was found that high baseline level of PLTs, lymphocytes, CRP, SII, NLR, PLR, LCR, LMR, HGB, HCT, MCH, ALB, AGR, TP, HDL-C, TG, ApoA-I, Apo-B, and LDH; and low baseline level of PLT, CRP, monocytes, SII, NLR, PLR, LCR, LMR, HGB, HCT, MCV, GLOB, TP, CHO, HDL-C, LDL-C, ApoA-I, and LDH in peripheral blood indicated better DFS prognosis for the post-operative ICU group with both HR value < 1 (Supplementary Figure S1B, Supplementary Table S7). In addition, high baseline level of WBCs, lymphocytes, CRP, PLR, LCR, LMR, HGB, HCT, MCH, ALB, GLOB, AGR, TP, CHO, HDL-C, TG, ApoA-I, and Apo-B; and low baseline level of PLTs, CRP, monocytes, SII, NLR, PLR, LCR, LMR, HGB, HCT, MCV, ALB, GLOB, AGR, TP, CHO, LDL-C, ApoA-I, and LDH in peripheral blood indicated longer OS prognosis for the post-operative ICU group with both HR value < 1 (Figure 3B, Supplementary Table S7).

For the post-operative levels of hematological indications, it was found that high levels of WBCs, neutrophils, lymphocytes, CRP, monocytes, SII, NLR, PLR, LMR, HGB, HCT, MCV, MCH, AGR, TP, CHO, HDL-C, LDL-C, TG, ApoA-I, Apo-B, and LDH; and low levels of RBCs, WBCs, neutrophils, PLTs, lymphocytes, monocytes, NLR, PLR, LCR, LMR, HCT, MCV, ALB, GLOB, AGR, TP, CHO, LDL-C, TG, ApoA-I, and LDH in peripheral blood at the post-operative stage indicated a better DFS prognosis for the post-operative ICU group with both HR value < 1 (Supplementary Figure S1B, Supplementary Table S8). In addition, high levels of WBCs, neutrophils, lymphocytes, CRP, monocytes, SII, NLR, PLR, LCR, LMR, HGB, HCT, MCH, ALB, AGR, TP, CHO, HDL-C, TG, ApoA-I, Apo-B, and LDH; and low levels of RBCs, WBCs, neutrophils, PLTs, lymphocytes, CRP, monocytes, SII, NLR, PLR, LCR, LMR, HGB, HCT, MCV, MCH, ALB, GLOB, TP, CHO, HDL-C, LDL-C, TG, and ApoA-I in peripheral blood indicated a longer OS prognosis for the post-operative ICU group with both HR value < 1 (Figure 3B, Supplementary Table S8).

For the fluctuating levels of hematological indicators, we found that elevated fluctuating levels of PLTs, lymphocytes, CRP, SII, NLR, PLR, LCR, LMR, HGB, HCT, MCH, ALB, AGR, TP, HDL-C, TG, ApoA-I, Apo-B, and LDH and reduced fluctuating levels of PLTs, CRP, monocytes, SII, NLR, PLR, LCR, LMR, HGB, HCT, MCV, GLOB, TP, CHO, HDL-C, LDL-C, ApoA-I, and LDH in peripheral blood indicated a superior DFS prognosis for the post-operative ICU group with both HR value < 1 (Supplementary Figure S1B, Supplementary Table S6). In addition, elevated fluctuating levels of WBCs, lymphocytes, CRP, PLR, LCR, LMR, HGB, HCT, MCH, ALB, GLOB, AGR, TP, CHO, HDL-C, TG, ApoA-I, and Apo-B; and reduced fluctuating peripheral blood levels of PLTs, CRP, monocyte, SII, NLR, PLR, LCR, LMR, HGB, HCT, MCV, ALB, GLOB, AGR, TP, CHO, LDL-C, ApoA-I, and LDH suggested a favorable OS prognosis for the post-operative ICU admission group with both HR value < 1 (Figure 3B, Supplementary Table S6).

Analysis of the other subgroups of hematological parameters showed no significant differences in prognosis between the ICU and non-ICU groups.

### 3.6. Survival Analysis Using Cox Univariate and Multivariate Analyses

Supplementary Figures S2–S5 detail the prognostic factors identified using univariate and multivariate analyses. In the univariate analyses, for all patients, the risk factors relevant to DFS were gender; age; T, N, M, stage; smoking history; drinking history; radiotherapy; adjuvant immunotherapy; pathological type; surgical method; the baseline levels of RBCs, WBCs, PLTs, monocytes, GLOB, LDH, ALB, CRP, ApoA-I, LCR, PLR, and LMR; the post-operative levels of WBCs, neutrophils, LDH, ALB, CRP, TG, ApoA-I, NLR, PLR, and SII; and the fluctuating levels of WBCs neutrophils, PLTs, monocytes, LDH, ALB, CRP, ApoA-I, NLR, LCR, PLR, and SII (Supplementary Figure S2). In all patients, the risk factors relevant to OS were gender; age; T, N, M, and stage; smoking history; drinking history; family history of cancer; radiotherapy; adjuvant immunotherapy; pathological type; surgical method; the baseline levels of RBCs, WBCs, neutrophils, PLTs, monocytes, HGB, HCT, TP, LDH, ALB, CRP, ApoA-I, NLR, LCR, PLR, and SII; the post-operative levels of RBCs, WBCs, PLTs, monocytes, HGB, HCT, LDH, ALB, CRP, CHO, HDL-C, ApoA-I, LCR, PLR, and LMR; and the fluctuating levels of HGB, HCT, MCH, LDH, CHO, and LCR (Supplementary Figure S3). Moreover, patients who were female; age ≥ 60; had T1, N0, M0, stage I disease; were non-smokers; were non-drinkers; did not receive radiotherapy or adjuvant immunotherapy; had adenocarcinoma; received wedge resection; had lower baseline levels of RBCs, WBCs, PLTs, monocytes, LDH, CRP, and PLR; higher baseline levels of ApoA-I, ALB, and LCR; lower post-operative LDH levels; and lower fluctuating levels of LDH and LCR exhibited superior DFS and OS (all *p* < 0.05).

According to the multivariate assessment, age, T, N, and M, disease stage, whether the patient received radiotherapy or adjuvant immunotherapy, whether adenocarcinoma is present, the baseline levels of LMR and the post-operative levels of LDH, CRP, and ApoA-I were found to be independent indicators for DFS (Supplementary Figure S4). In addition, gender, age, T, N, M, and disease stage, smoking history, family history of cancer, whether received radiotherapy and adjuvant immunotherapy, the baseline levels of RBCs, LDH, ALB, CRP, and LCR, the post-operative levels of HCT, LDH, ApoA-I, and LMR, and the fluctuating level of MCH were found to be independent indicators for OS (Supplementary Figure S5). Generally, with T1, N0, M0 or stage I disease, no receipt of radiotherapy or adjuvant immunotherapy, a lower post-operative level of LDH independently predicted better DFS and OS (all *p* < 0.05).

### 3.7. Model Development, Validation, and Comparison

To identify those patients who could benefit from admission to the ICU, we constructed four predictive models (SVM, Catboost, ANN, and KNN) using clinically available clinical features and hematological parameters data at the baseline stage. Cox regression analysis found that most of these clinical features were closely related to cancer prognosis, indicating the feasibility of modeling using these indicators. We randomly divided the study population into a training set and an internal validation set, with a 7:3 ratio, and emphasized the importance of all clinical indicators for each model in the training cohort. Each of the four models was constructed on the most appropriate indicators, as shown in Figure 4B–E and Supplementary Table S1. To verify the reliability of these selected metrics, they were also applied to the internal validation cohort.

**Figure 5:**
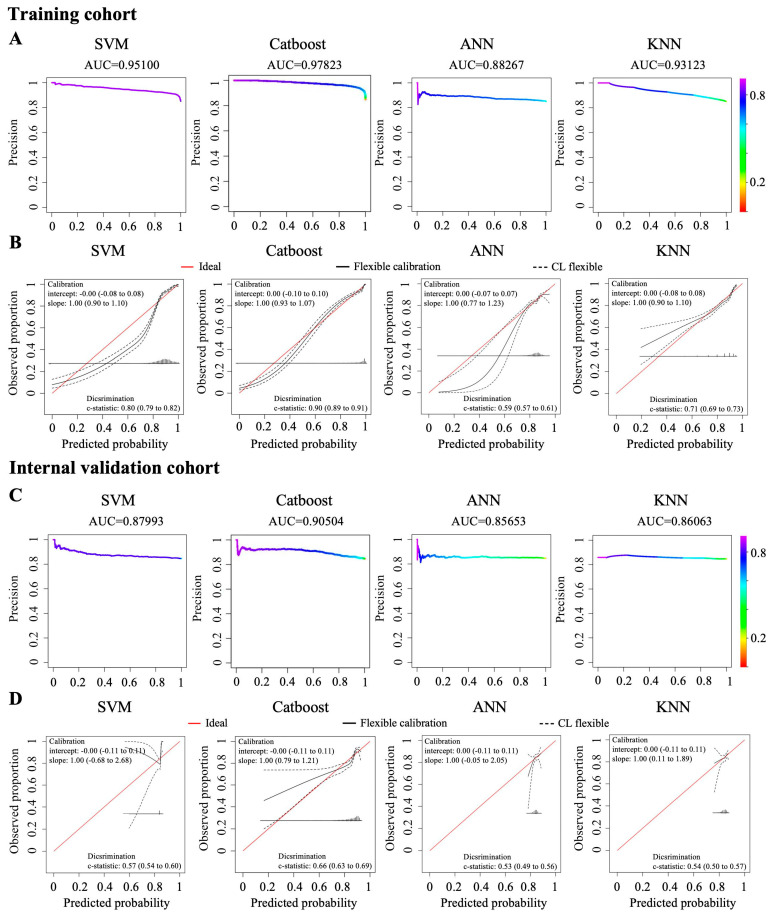
Predictive performance of the four models using clinical characteristics and baseline hematological parameters. The AUC of the four predictive models based on clinical characteristics and fluctuating hematological parameters in the training cohort (**A**) and the internal validation cohort (**C**). Calibration plot of the four predictive models in the training cohort (**B**) and the internal validation cohort (**D**).

For the training cohort, based on the clinical characteristics and the level of baseline blood indicators, the Catboost and SVM models showed excellent discrimination, with AUC values of 0.97823 and 0.95100, respectively; however, the ANN and KNN models showed poorer discrimination, with AUC values of 0.88267 and 0.93123, respectively (Figure 5A, Supplementary Table S9).

For the internal validation cohort, based on the clinical characteristics and the baseline blood indicator levels, the Catboost model also showed more prominent discrimination than the SVM, ANN, and KNN models (AUCs = 0.90504, 0.87993, 0.85653, and 0.86063, respectively. (Figure 5C, Supplementary Table S9).

In the Catboost model, across the whole spectrum of predicted risks, there was a generally good alignment of the observed and predicted risks according to the calibration plots, although with some minor overestimation or underestimation (Figure 5B,D). Therefore, the predictive power of the Catboost model was statistically superior to that of the other models in the training and internal validation cohorts.

### 3.8. Model Performance and Decision Curve Analysis

**Figure 6:**
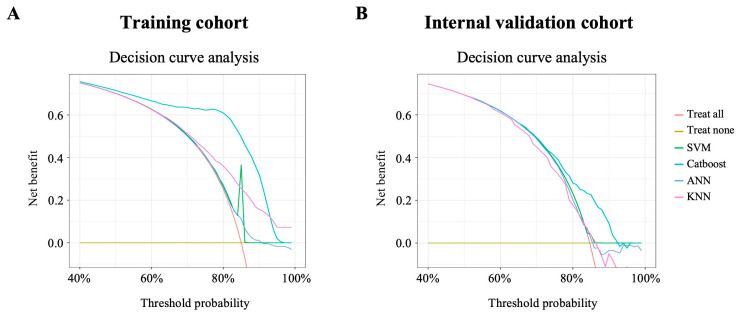
Decision curve analysis of the models using baseline hematological parameters. Decision curve analysis based on baseline hematological parameters in the training cohort (**A**) and the internal validation cohort (**B**).

Based on the clinical characteristics and the level of baseline hematological parameters, clinical decision curves for the training cohort indicated that after accounting for competing risks, the Catboost model indicated a generally better benefit, whereas the SVM model showed the least clinical utility (Figure 6A). The same evaluation method was applied to the internal validation cohort, which also showed that the Catboost model was the optimal predictor of ICU admission in patients with cancer after surgery (Figure 6B).

## 4. Discussion

Herein, we observed that ICU admission after surgery was associated with superior survival in patients with cancer. We then explored the potential effects of clinical characteristics and hematological parameters on the decision for ICU admission. Subsequently, a machine learning model to predict ICU admission following intent to cure surgery in patients with cancer, which employed routinely acquired blood test results and basic clinical data, was developed. The machine learning models, developed based on clinical data and baseline hematological parameters, were SVM, Catboost, ANN, and KNN. Importantly, ICU admission after surgery was associated with superior survival in patients with cancer in most of the clinical data subgroup analyses. This emphasized the utility of clinical data to identify and predict patients with cancer who might prioritize ICU admission in terms of longer survival and could help with clinical decision making after surgery. Compared with the other three prediction models, Catboost showed a comparable predictive performance for ICU admission, but with an outstanding AUC.

Recently, cancer outcomes have improved because of advances in therapeutics. Consequently, more patients with cancer have become candidates for ICU admission. Many surgical cancer patients suffer from complications, frequently resulting in poor outcomes. However, there have been few reports on post-operative management strategies. A few previous studies have discussed whether patients with cancer who are admitted to the ICU experience a survival benefit, but the results are inconclusive [16,17,18]. In 2005, an initial prospective study evaluated the outcomes of patients with cancer who were candidates for ICU admission. The authors concluded that both the somewhat good survival of too-sick patients and the increased deaths among too-well ICU-admitted patients suggested a requirement to formulate policy to decide on admissions [19]. Another study on cancer also suggested that patients with better progression-free status could benefit from being admitted to the ICU [20]. However, a large retrospective study showed that the majority of Medicare-enrolled patients with cancer who were treated in an ICU died during the 6 months after admission [21]. Moreover, in patients with acute myeloid leukemia, ICU admission was associated with high mortality and increased expense that was proportional to the comorbidity burden [22]. Therefore, previous studies have not well defined the benefits of ICU admission in patients with cancer. These studies mainly focused on the benefits of ICU admission in all-stage cancer patients with serious complications, but did not discuss the benefits of ICU admission after surgery for early-stage cancer. Hence, clinical data on the benefits of ICU admission after cancer surgery are very limited. In our study, we verified the current prioritization of ICU admission in a large cohort of patients with cancer and further explored the effect of clinical characteristics and hematological parameters on ICU admission.

ICU wards have limited capacity and are costly; however, there is little objective data regarding the post-surgical allocation of ICU beds. In contrast to most previous studies on predictors for post-operative ICU stay, which were carried out for specific surgeries, our results are applicable to a wider range of surgical cancer populations. The analytical model Surgical Risk Preoperative Assessment System (SURPAS) employs eight pre-operative variables for prediction and can be utilized for pre-operative planning and the allocation of limited numbers of ICU beds [23]. Another group developed a pre-surgery prediction model employing data from a large and broad surgical population: the Combined Assessment of Risk Encountered in Surgery (CARES) model, to be used for post-operative ICU allocation [24]. It has been reported that these models only applied clinical feature data and did not consider the effect of hematological parameters on prognosis. However, in our study, we considered the clinical characteristics and hematological parameters and carried out subgroup analysis based on them. Our analysis showed that ICU admission prioritizes patients with several clinical characteristics and indicated the level of hematological parameters that might serve as predictors for ICU admission. Moreover, we constructed four learning models to predict ICU admission. Among them, the Catboost model, which includes clinical data and the level of hematological parameters at baseline stage, all of which are derived preoperatively and are available from a wide-ranging surgical population, represents an attractive tool for health care professionals. Indeed, no previous study has provided a specific model for post-operative ICU admission in patients with cancer.

We developed the predictive machine learning model using clinical data and the level of baseline hematological parameters; therefore, its main strength is the use of routinely acquired clinical data as input, thereby minimizing the extra time and expenditure needed to prepare and process the data. All features used in Catboost are routinely collected in hospitals and can be accessed via patient clinical records, making our strategy noninvasive, cost-effective, and highly accessible, representing Catboost’s core benefits. Additionally, while there have been some reports on the outcomes of cancer patients after ICU admission, to the best of our knowledge, no systematic attempts have been made to summarize their findings or evaluate the quality of existing evidence, particularly to reduce the harm associated with unplanned ICU admissions. Our study demonstrated that patients undergoing cancer surgery followed by ICU admission achieved favorable outcomes by implementing early ICU management strategies, reducing the incidence of common post-operative complications, and enhancing their post-operative recovery process. Most importantly, by establishing a predictive model to identify the characteristics of patients who would currently prioritize ICU admission, our study provides more detailed information to guide surgeons’ decision making regarding ICU hospitalization for patients with cancer after surgery. Through data-driven analysis, we provided more accurate and personalized management solutions for patients after cancer surgery and promoted the progress of medical technology. However, our model provides a pre-operative risk probability based solely on hematologic and basic clinical data. It is explicitly designed as a screening tool for resource planning, not as a replacement for perioperative clinical judgment. The final ICU admission decision must integrate our model’s baseline risk assessment with intraoperative findings, including surgical complexity, blood loss, hemodynamic stability, and anesthesiologist evaluation, which our model cannot capture.

There are several limitations to this study. First, the ICU admission and non-ICU admission datasets were unbalanced, necessitating an external test set for verification. Second, despite attempting to mitigate the sample size limitation via test dataset validation, the small sample size might have resulted in model overfitting. Our research is a Phase I machine learning study (development and internal validation only), with external validation clearly identified as essential. Furthermore, the intraoperative situation of the patients represented a confounding bias and contributed to the reported heterogeneity. Moreover, the results might have been subjected to potential bias, confounding factors, and missing data because of the retrospective nature of the study.

Herein, we provided insights regarding the predictive value of post-operative ICU admission in surgery-treated patients with cancer. We also analyzed the effect of clinical and hematological parameters on ICU admission in detail and established the most appropriate model to identify which patients are suitable for ICU admission. Our results will assist clinicians in their decision making regarding post-operative ICU admission, aiming to provide more patients with cancer with affordable and high-quality critical care via ICU admission. Stratification techniques for patients with cancer might promote powerful methods to provide post-operative management strategies to reduce complications and improve survival. However, increased patient acuity, decreased ICU bed availability, and the patient’s financial condition have strained ICU’s post-operative capacity in some hospitals. In these particular settings, we recommend that patients with cancer after surgery be monitored and treated in a manner closely resembling ICU admission to improve their prognosis. Currently, little is known about the mechanism of the benefit derived from ICU admission after cancer surgery, and future prospective biomarker studies should employ complementary experimental avenues to investigate the underlying biological mechanisms of the model based on the identified clinical characteristics and hematological parameters. Our findings also support future exploration of the prospects of therapy based on hematological parameters, e.g., increased HGB, to improve patients’ response to treatment and consequently, the efficacy of cancer surgery in the long-term.

## 5. Conclusions

Post-operative ICU admission was associated with superior survival in patients with cancer. Most of the clinical characteristics and hematological parameters had an important effect on the ICU admission decision making. Our ensemble model, Catboost, based on clinical characteristics and baseline hematological parameters, could predict post-operative ICU admission and has good clinical application. Catboost could be used to stratify patients who might prioritize from ICU admission post-surgery under consideration for more rigorous treatment and is worthy of further clinical evaluation.

## Data Availability

The datasets used and analyzed in this study are available from the corresponding author upon reasonable request. The authenticity of this article has been validated by uploading the key raw data onto the Research Data Deposit public platform (www.researchdata.org.cn), with the approval RDD number as RDDA2026180491.

## Supplementary Materials

The following supporting information can be downloaded at: https://www.mdpi.com/article/10.3390/jcm15082898/s1, Figure S1: Effects of clinical characteristics and baseline/ post-operative/ fluctuating hematological parameters on DFS in patients the ICU and non-ICU groups; Figure S2: Cox univariate prognostic regression analysis of DFS for all clinical indicators; Figure S3: Cox univariate prognostic regression analysis of OS for all clinical indicators; Figure S4: Cox multivariate prognostic regression analysis of DFS for all clinical indicators; Figure S5: Cox multivariate prognostic regression analysis of OS for all clinical indicators; Table S1: Baseline characteristics of the study population; Table S2: The impact of clinical characteristics on DFS and OS in patients with and without ICU admission; Table S3: The alterations of hematologic parameters in all patients; Table S4: The cut-off values of hematologic parameters in all patients; Table S5: The impact of dynamic changes in hematologic parameters on DFS and OS in patients with and without ICU admission; Table S6: The impact of baseline hematologic parameters on DFS and OS in patients with and without ICU admission; Table S7: The impact of post-surgery hematologic parameters on DFS and OS in patients with and without ICU admission; Table S8: Importance of all clinical indicators for all four models; Table S9: Summary performance metrics of all four models based on baseline hematologic parameters.

## Abbreviations

ICU, intensive care unit; AUC, area under the curve; IC, intensive care; DFS, disease-free survival; OS, overall survival; EDTA, ethylene diamine tetraacetic acid; RBC, red blood cell; WBC, white blood cell; PLT, platelet; CRP, C-reactive protein; SII, immune-inflammation index; NLR, neutrophil/lymphocyte ratio; PLR, platelet/lymphocyte ratio; LCR, lymphocyte-to-C-reactive protein ratio; LMR, lymphocyte/monocyte ratio; HGB, hemoglobin; HCT, hematocrit; MCV, mean corpuscular volume; MCH, mean corpuscular hemoglobin; ALB, albumin; GLOB, globulin; AGR, ALB-to-GLOB ratio; TP, total protein; CHO, cholesterol; HDL-C, high-density lipoprotein-cholesterol; LDL-C, low-density lipoprotein-cholesterol; TG, triglycerides; ApoA-I, apolipoprotein A-1; Apo-B, apolipoprotein B; LDH, lactate dehydrogenase; SVM, support vector machines; ANN, artificial neural network; Catboost, categorical boosting; KNN, K-Nearest Neighbor; PR, precision–Recall; CI, confidence interval; PI, prediction interval; DCA, decision curve analysis.
