# Supplementary material for: A Machine Learning Model to Predict Post-Operative Intensive Care Unit Admission in Patients with Cancer Based on Clinical Characteristics and Hematologic Parameters Data
Source: J Clin Med. 2026 Apr 10;15(8):2898. doi: 10.3390/jcm15082898 (PMC13115788; doi:10.3390/jcm15082898)
Supplement: Supplementary file 1 [file jcm-15-02898-s001.zip › jcm-4199233-supplementary.pdf]

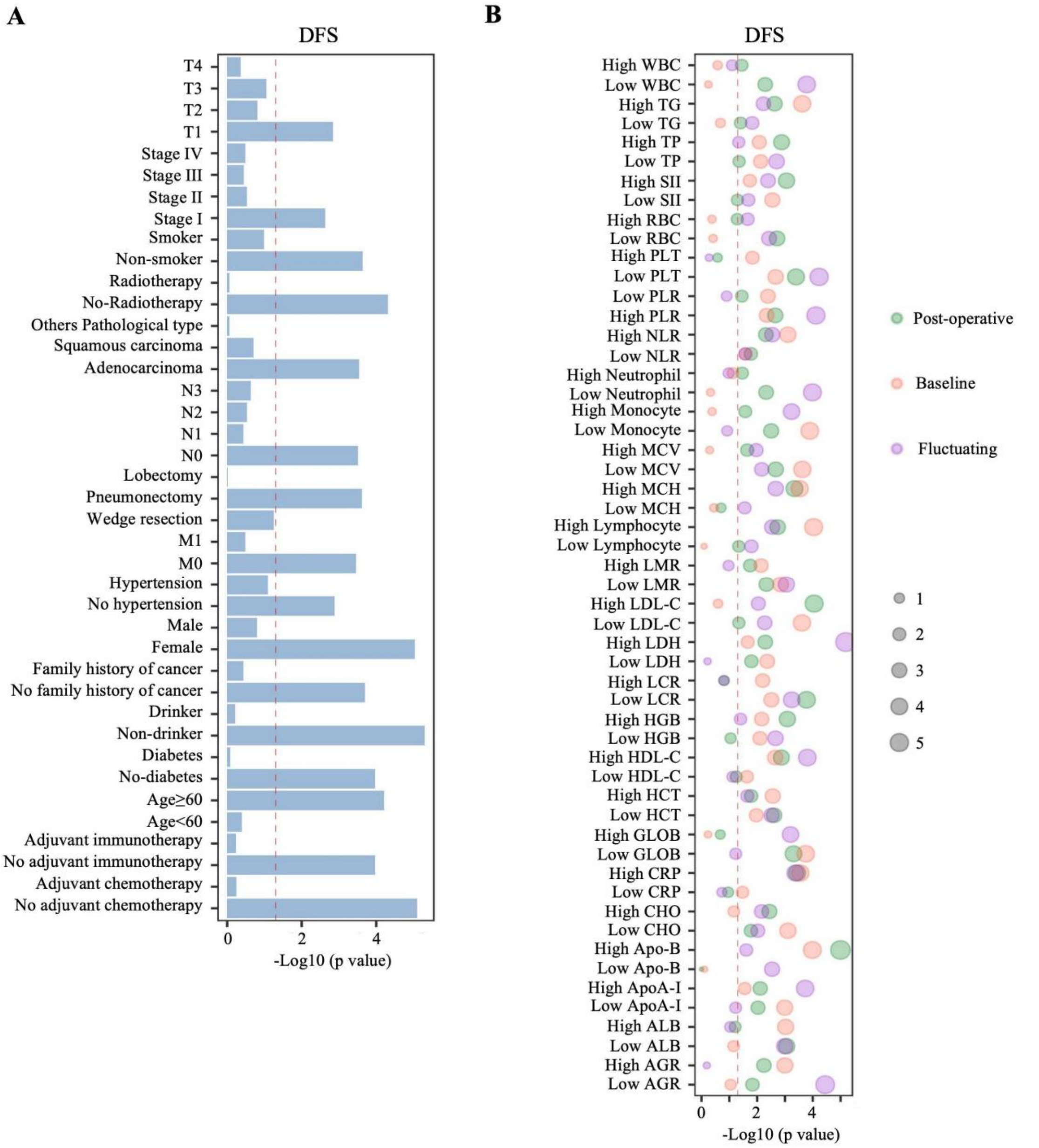

Supplementary Figure S1. Effects of clinical characteristics and baseline/ post-operative/ fluctuating hematological parameters on DFS in patients the ICU and non-ICU groups. (A) Subgroup analysis showing a superior DFS in the ICU group compared with that in the non-ICU group for the above clinical characteristics. (B) Subgroup analysis showing a superior DFS in the ICU group compared with that in the non-ICU group for the above hematologic parameters.

Group	Feature	HR (CI 95%)	p value
Baseline	Gender	1.417 (1.257 − 1.597)	***
Baseline	Age	0.819 (0.73 − 0.919)	**
Baseline	T	1.885 (1.676 − 2.119)	***
Baseline	N	2.777 (2.471 − 3.12)	***
Baseline	M	1.804 (1.276 − 2.551)	**
Baseline	Stage	2.969 (2.616 − 3.369)	***
Baseline	Smoking history	1.452 (1.296 − 1.628)	***
Baseline	Drinking history	1.341 (1.178 − 1.527)	***
Baseline	Diabetes	0.814 (0.659 − 1.007)	ns
Baseline	Hypertension	0.861 (0.734 − 1.01)	ns
Baseline	Family history of cancer	0.88 (0.763 − 1.014)	ns
Baseline	Radiotherapy	4.094 (3.528 − 4.751)	***
Baseline	Adjuvant chemotherapy	1.036 (0.901 − 1.192)	ns
Baseline	Adjuvant immunotherapy	2.894 (2.408 − 3.479)	***
Baseline	Adenocarcinoma	0.761 (0.67 − 0.863)	***
Baseline	Squamous carcinoma	1.36 (1.181 − 1.566)	***
Baseline	Others pathological type	1.1 (0.895 − 1.35)	ns
Baseline	Wedge resection	0.666 (0.585 − 0.757)	***
Baseline	Pneumonectomy	1.344 (1.198 − 1.507)	***
Baseline	Lobectomy	1.093 (0.947 − 1.262)	ns
Baseline	RBC	1.131 (1.051 − 1.218)	**
Baseline	WBC	1.037 (1.023 − 1.052)	***
Baseline	Neutrophil	1.002 (0.997 − 1.007)	ns
Baseline	Lymphocyte	0.987 (0.945 − 1.031)	ns
Baseline	PLT	1.001 (1 − 1.001)	***
Baseline	Monocyte	1.068 (1.019 − 1.119)	**
Baseline	HGB	0.997 (0.994 − 1)	ns
Baseline	HCT	1.001 (0.997 − 1.005)	ns
Baseline	MCV	1 (0.999 − 1.001)	ns
Baseline	MCH	0.993 (0.974 − 1.012)	ns
Baseline	TP	0.998 (0.987 − 1.009)	ns
Baseline	GLOB	1 (1 − 1.001)	*
Baseline	AGR	1.003 (0.995 − 1.011)	ns
Baseline	LDH	1.001 (1.001 − 1.002)	***
Baseline	ALB	0.973 (0.962 − 0.985)	***
Baseline	CRP	1.002 (1.001 − 1.003)	***
Baseline	CHO	0.998 (0.986 − 1.011)	ns
Baseline	TG	1.014 (0.994 − 1.034)	ns
Baseline	HDL-C	0.969 (0.883 − 1.062)	ns
Baseline	LDL-C	0.987 (0.947 − 1.03)	ns
Baseline	ApoA-I	0.607 (0.481 − 0.765)	***
Baseline	Apo-B	1.082 (0.846 − 1.383)	ns
Baseline	NLR	1.002 (0.994 − 1.01)	ns
Baseline	LCR	0.945 (0.925 − 0.965)	***
Baseline	PLR	1.001 (1 − 1.001)	*
Baseline	LMR	0.912 (0.885 − 0.939)	***
Baseline	SII	1 (1 − 1)	ns
Post-operative	RBC	1.007 (0.96 − 1.057)	ns
Post-operative	WBC	0.951 (0.934 − 0.967)	***
Post-operative	Neutrophil	0.94 (0.924 − 0.957)	***
Post-operative	Lymphocyte	1.002 (0.989 − 1.016)	ns
Post-operative	PLT	1 (1 − 1)	ns
Post-operative	Monocyte	0.978 (0.895 − 1.069)	ns
Post-operative	HGB	1 (0.998 − 1.001)	ns
Post-operative	HCT	1 (1 − 1.001)	ns
Post-operative	MCV	1.001 (0.997 − 1.005)	ns
Post-operative	MCH	0.997 (0.979 − 1.017)	ns
Post-operative	TP	1 (1 − 1)	ns
Post-operative	GLOB	1 (0.999 − 1.001)	ns
Post-operative	AGR	1.003 (0.993 − 1.013)	ns
Post-operative	LDH	1.001 (1 − 1.001)	***
Post-operative	ALB	1.003 (1.001 − 1.006)	*
Post-operative	CRP	0.995 (0.994 − 0.997)	***
Post-operative	CHO	1.003 (0.996 − 1.011)	ns
Post-operative	TG	1.012 (1.001 − 1.024)	*
Post-operative	HDL-C	0.995 (0.967 − 1.024)	ns
Post-operative	LDL-C	1.001 (0.994 − 1.008)	ns
Post-operative	ApoA-I	1.25 (1.14 − 1.371)	***
Post-operative	Apo-B	1.002 (0.968 − 1.037)	ns
Post-operative	NLR	0.955 (0.942 − 0.967)	***
Post-operative	LCR	0.994 (0.976 − 1.012)	ns
Post-operative	PLR	0.999 (0.998 − 0.999)	***
Post-operative	LMR	0.999 (0.992 − 1.007)	ns
Post-operative	SII	1 (1 − 1)	***
Fluctuating	RBC	0.958 (0.912 − 1.007)	ns
Fluctuating	WBC	0.95 (0.941 − 0.96)	***
Fluctuating	Neutrophil	0.995 (0.993 − 0.998)	***
Fluctuating	Lymphocyte	1.003 (0.993 − 1.014)	ns
Fluctuating	PLT	0.999 (0.999 − 1)	**
Fluctuating	Monocyte	0.942 (0.897 − 0.989)	*
Fluctuating	HGB	1.001 (1 − 1.002)	ns
Fluctuating	HCT	1 (1 − 1.001)	ns
Fluctuating	MCV	1 (0.999 − 1.001)	ns
Fluctuating	MCH	1.015 (0.983 − 1.048)	ns
Fluctuating	TP	1 (1 − 1)	ns
Fluctuating	GLOB	1 (0.999 − 1)	ns
Fluctuating	AGR	1 (0.992 − 1.008)	ns
Fluctuating	LDH	1.001 (1 − 1.001)	**
Fluctuating	ALB	1.004 (1.002 − 1.007)	***
Fluctuating	CRP	0.997 (0.997 − 0.998)	***
Fluctuating	CHO	1.003 (0.996 − 1.01)	ns
Fluctuating	TG	1.008 (0.995 − 1.022)	ns
Fluctuating	HDL-C	1.003 (0.988 − 1.018)	ns
Fluctuating	LDL-C	1.002 (0.996 − 1.007)	ns
Fluctuating	ApoA-I	1.337 (1.247 − 1.434)	***
Fluctuating	Apo-B	1 (0.963 − 1.038)	ns
Fluctuating	NLR	0.992 (0.989 − 0.995)	***
Fluctuating	LCR	1.004 (1.001 − 1.008)	*
Fluctuating	PLR	0.999 (0.999 − 0.999)	***
Fluctuating	LMR	1.002 (1 − 1.005)	ns
Fluctuating	SII	1 (1 − 1)	***

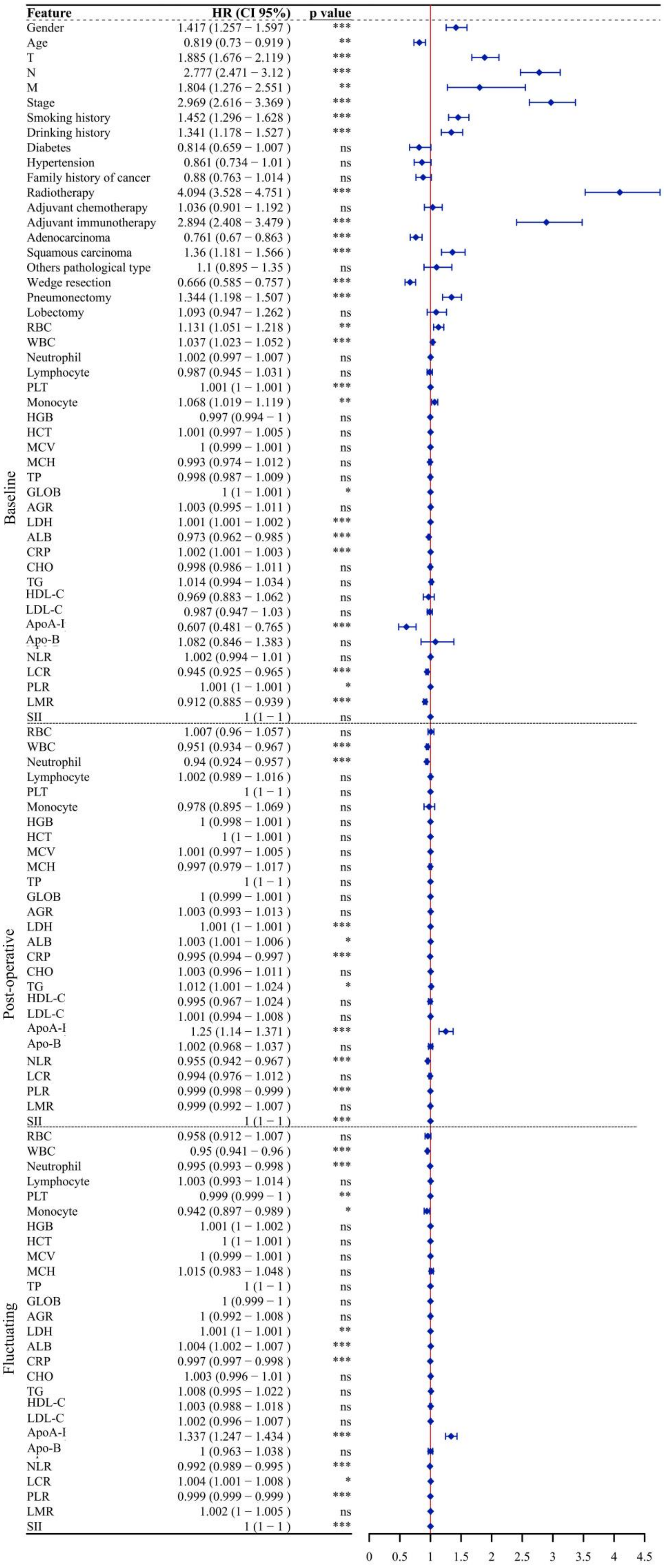

Supplementary Figure S2. Cox univariate prognostic regression analysis of DFS for all clinical indicators. Gender, Male vs. Female; Age, ≥ 60 vs. < 60; T, T2-4 vs. T1; N, N1-3 vs. N0; M, M1 vs. M0; Stage, Stage II-IV vs. Stage I; Smoking history, smoking vs. non-smoking; Drinking history, drinking vs. non-drinking; Diabetes, diabetes vs. non-diabetes; Hypertension, hypertension vs. non-hypertension; Family history of cancer, yes vs. no; Radiotherapy, received radiotherapy vs. not received radiotherapy; Adjuvant chemotherapy, received adjuvant chemotherapy vs. not received adjuvant chemotherapy; Adjuvant immunotherapy, received adjuvant immunotherapy vs. not received adjuvant immunotherapy; Adenocarcinoma, adenocarcinoma vs. non-adenocarcinoma; Squamous carcinoma, squamous carcinoma vs. non-squamous carcinoma; Others, others pathological type vs. non-others pathological type; Wedge resection, perform wedge resection vs. not perform wedge resection; Pneumonectomy, perform pneumonectomy vs. not perform pneumonectomy; Lobectomy, perform lobectomy vs. not perform lobectomy.

	Feature	HR (CI 95%)	p value
Baseline	Gender	1.871 (1.68 – 2.085)	***
	Age	1.389 (1.251 – 1.542)	***
	T	2.514 (2.262 – 2.793)	***
	N	3.176 (2.865 – 3.521)	***
	M	2.425 (1.848 – 3.182)	***
	Stage	4.298 (3.805 – 4.855)	***
	Smoking history	1.869 (1.694 – 2.062)	***
	Drinking history	1.414 (1.268 – 1.576)	***
	Diabetes	0.983 (0.828 – 1.167)	ns
	Hypertension	0.879 (0.763 – 1.011)	ns
	Family history of cancer	0.742 (0.653 – 0.844)	***
	Radiotherapy	1.702 (1.442 – 2.01)	***
	Adjuvant chemotherapy	0.952 (0.842 – 1.075)	ns
	Adjuvant immunotherapy	2.122 (1.796 – 2.508)	***
	Adenocarcinoma	0.549 (0.496 – 0.608)	***
	Squamous carcinoma	1.899 (1.7 – 2.122)	***
	Others pathological type	1.257 (1.068 – 1.48)	**
	Wedge resection	0.592 (0.527 – 0.664)	***
	Pneumonectomy	1.45 (1.313 – 1.602)	***
	Lobectomy	1.114 (0.984 – 1.262)	ns
	RBC	1.114 (1.047 – 1.186)	**
	WBC	1.055 (1.046 – 1.064)	***
	Neutrophil	1.004 (1.001 – 1.007)	**
	Lymphocyte	0.987 (0.95 – 1.026)	ns
	PLT	1.001 (1 – 1.001)	***
	Monocyte	1.089 (1.056 – 1.123)	***
	HGB	0.997 (0.994 – 1)	*
	HCT	0.985 (0.975 – 0.996)	**
	MCV	1 (0.999 – 1)	ns
	MCH	1.004 (0.988 – 1.021)	ns
	TP	0.985 (0.977 – 0.994)	**
	GLOB	1 (1 – 1.001)	ns
	AGR	1 (0.992 – 1.008)	ns
	LDH	1.001 (1.001 – 1.002)	***
	ALB	0.945 (0.938 – 0.953)	***
	CRP	1.004 (1.003 – 1.004)	***
	CHO	1.003 (0.999 – 1.007)	ns
	TG	1.007 (0.985 – 1.031)	ns
	HDL-C	1.002 (0.987 – 1.018)	ns
	LDL-C	1 (0.989 – 1.012)	ns
	ApoA-I	0.374 (0.306 – 0.458)	***
	Apo-B	1.031 (0.835 – 1.274)	ns
	NLR	1.005 (1.001 – 1.009)	*
	LCR	0.889 (0.868 – 0.91)	***
	PLR	1.001 (1 – 1.001)	***
	LMR	1 (1 – 1.001)	ns
	SII	1 (1 – 1)	***
Post-operative	RBC	1.042 (1.002 – 1.085)	*
	WBC	1.005 (1.002 – 1.008)	**
	Neutrophil	1 (0.999 – 1)	ns
	Lymphocyte	1.002 (0.99 – 1.014)	ns
	PLT	1 (1 – 1.001)	**
	Monocyte	1.05 (1.027 – 1.073)	***
	HGB	0.989 (0.987 – 0.992)	***
	HCT	0.955 (0.947 – 0.963)	***
	MCV	0.996 (0.991 – 1.002)	ns
	MCH	0.987 (0.971 – 1.003)	ns
	TP	1 (1 – 1)	ns
	GLOB	1 (0.999 – 1.001)	ns
	AGR	1.005 (0.997 – 1.013)	ns
	LDH	1.001 (1.001 – 1.001)	***
	ALB	0.967 (0.96 – 0.975)	***
	CRP	1.002 (1.001 – 1.003)	***
	CHO	0.901 (0.861 – 0.942)	***
	TG	0.978 (0.945 – 1.012)	ns
	HDL-C	0.563 (0.488 – 0.65)	***
	LDL-C	0.999 (0.988 – 1.009)	ns
	ApoA-I	0.417 (0.352 – 0.494)	***
	Apo-B	0.972 (0.876 – 1.079)	ns
	NLR	1 (0.999 – 1)	ns
	LCR	0.905 (0.878 – 0.933)	***
	PLR	1.001 (1 – 1.001)	***
	LMR	0.893 (0.872 – 0.916)	***
	SII	1 (1 – 1)	ns
Fluctuating	RBC	0.999 (0.959 – 1.04)	ns
	WBC	0.99 (0.978 – 1.002)	ns
	Neutrophil	0.999 (0.994 – 1.003)	ns
	Lymphocyte	1.003 (0.994 – 1.013)	ns
	PLT	1 (1 – 1)	ns
	Monocyte	1.024 (0.987 – 1.061)	ns
	HGB	0.999 (0.998 – 1)	*
	HCT	0.997 (0.994 – 0.999)	**
	MCV	1 (1 – 1.001)	ns
	MCH	0.945 (0.92 – 0.971)	***
	TP	1 (1 – 1)	ns
	GLOB	1 (0.999 – 1)	ns
	AGR	1.003 (0.996 – 1.01)	ns
	LDH	1.001 (1 – 1.001)	***
	ALB	1.001 (0.998 – 1.004)	ns
	CRP	1 (0.999 – 1.001)	ns
	CHO	0.996 (0.993 – 0.999)	*
	TG	0.986 (0.97 – 1.002)	ns
	HDL-C	0.991 (0.981 – 1.001)	ns
	LDL-C	0.999 (0.992 – 1.006)	ns
	ApoA-I	1.013 (0.98 – 1.046)	ns
	Apo-B	0.958 (0.819 – 1.121)	ns
	NLR	0.999 (0.994 – 1.004)	ns
	LCR	1.004 (1.001 – 1.007)	*
	PLR	1 (1 – 1.001)	ns
	LMR	1 (1 – 1)	ns
	SII	1 (1 – 1)	ns

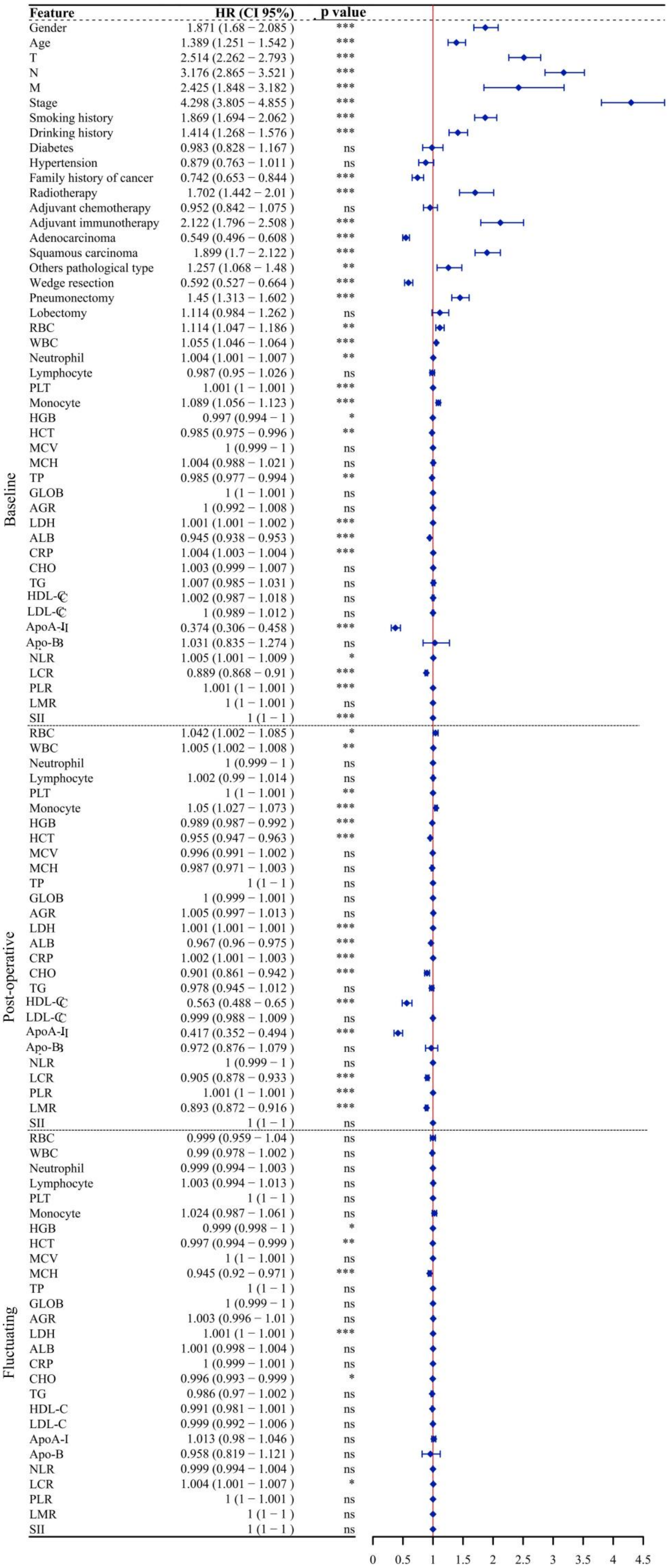

Supplementary Figure S3. Cox univariate prognostic regression analysis of OS for all clinical indicators. Gender, Male vs. Female; Age, ≥ 60 vs. <60; T, T2-4 vs. T1; N, N1-3 vs. N0; M, M1 vs. M0; Stage, Stage II-IV vs. Stage I; Smoking history, smoking vs. non-smoking; Drinking history, drinking vs. non-drinking; Diabetes, diabetes vs. non-diabetes; Hypertension, hypertension vs. non-hypertension; Family history of cancer, yes vs. no; Radiotherapy, received radiotherapy vs. not received radiotherapy; Adjuvant chemotherapy, received adjuvant chemotherapy vs. not received adjuvant chemotherapy; Adjuvant immunotherapy, received adjuvant immunotherapy vs. not received adjuvant immunotherapy; Adenocarcinoma, adenocarcinoma vs. non-adenocarcinoma; Squamous carcinoma, squamous carcinoma vs. non-squamous carcinoma; Others, others pathological type vs. non-others pathological type; Wedge resection, perform wedge resection vs. not perform wedge resection; Pneumonectomy, perform pneumonectomy vs. not perform pneumonectomy; Lobectomy, perform lobectomy vs. not perform lobectomy.

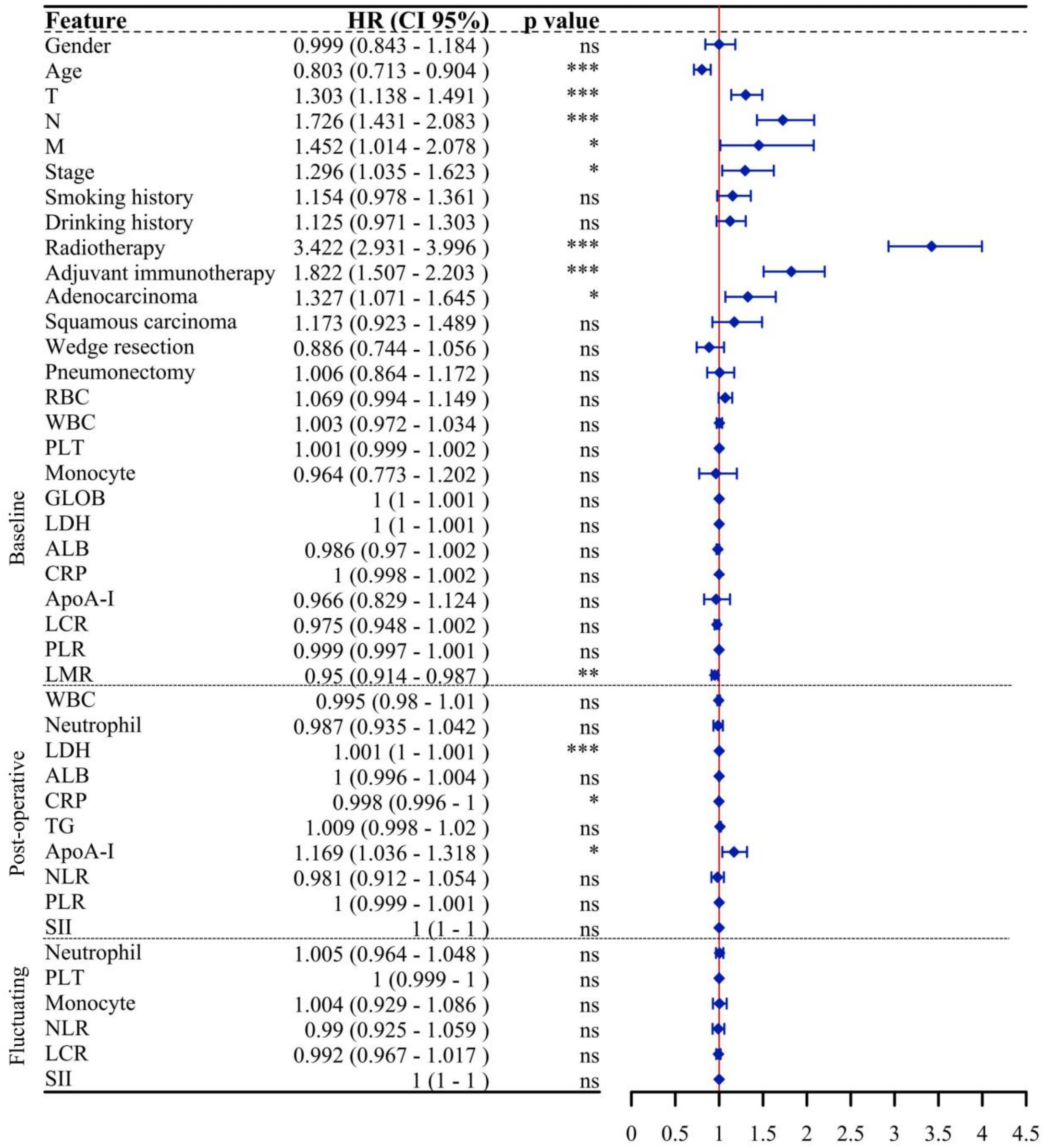

Supplementary Figure S4. Cox multivariate prognostic regression analysis of DFS for all clinical indicators. Gender, Male vs. Female; Age, >=60 vs. <60; T, T2-4 vs. T1; N, N1-3 vs. N0; M, M1 vs. M0; Stage, Stage II-IV vs. Stage I; Smoking history, smoking vs. non-smoking; Drinking history, drinking vs. non-drinking; No; Radiotherapy, received radiotherapy vs. not received radiotherapy; Adjuvant immunotherapy, received adjuvant immunotherapy vs. not received adjuvant immunotherapy; Adenocarcinoma, adenocarcinoma vs. non-adenocarcinoma; Squamous carcinoma, squamous carcinoma vs. non-squamous carcinoma; Wedge resection, perform wedge resection vs. not perform wedge resection; Pneumonectomy, perform pneumonectomy vs. not perform pneumonectomy.

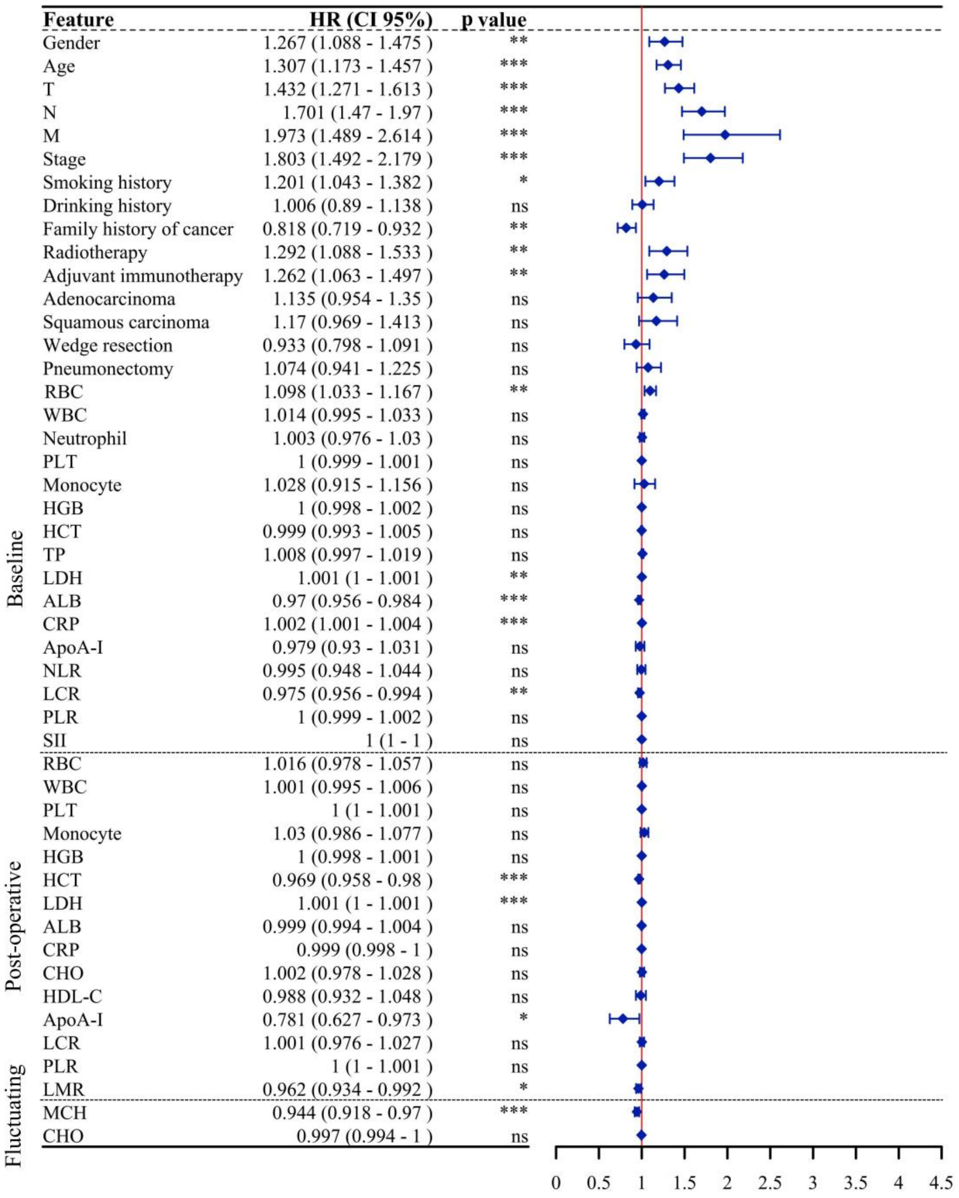

Supplementary Figure S5. Cox multivariate prognostic regression analysis of OS for all clinical indicators. Gender, Male vs. Female; Age, >=60 vs. <60; T, T2-4 vs. T1; N, N1-3 vs. N0; M, M1 vs. M0; Stage, Stage II-IV vs. Stage I; Smoking history, smoking vs. non-smoking; Drinking history, drinking vs. non-drinking; Family history of cancer, yes vs. no; Radiotherapy, received radiotherapy vs. not received radiotherapy; Adjuvant immunotherapy, received adjuvant immunotherapy vs. not received adjuvant immunotherapy; Adenocarcinoma, adenocarcinoma vs. non- adenocarcinoma; Squamous carcinoma, squamous carcinoma vs. non-squamous carcinoma; Wedge resection, perform wedge resection vs. not perform wedge resection; Pneumonectomy, perform pneumonectomy vs. not perform pneumonectomy.

Supplementary Table S1. Importance of all clinical indicators for all four models

Model	Cutoff	Variables
SVM	1 - AUC loss after permutation < 0.8	Adjuvant chemotherapy, Adjuvant immunotherapy, Age, ALB, Apo-B, CHO, CRP, Drinking history, Family history of cancer, Gender, HCT, HDL-C, HGB, Hypertension, LCR, LDH, LDL-C, LMR, MCH, Mode of operation, Monocyte, N, Neutrophil, NLR, Pathological type, PLR, PLT, Radiotherapy, RBC, SII, Smoking history, Stage, TP, TG, T, WBC
CatBoost	Importance > 1	Age, Mode of operation, Pathological type, T, N, Stage, Hypertension, Adjuvant chemotherapy, Adjuvant immunotherapy, RBC, WBC, Neutrophil, Lymphocyte, PLT, Monocyte, HGB, HCT, MCV, MCH, TP, GLOB, AGR, LDH, ALB, CRP, CHO, TG, HDL-C, LDL-C, ApoA-I, Apo-B, NLR, LCR, PLR, LMR, SII
ANN	Importance > 0.018	Neutrophil, SII, Pathological type, MCV, LMR, HDL–C, LDL–C, Radiotherapy, M, Diabetes, ALB, LCR, GLOB, Lymphocyte, ApoA–I, Adjuvant chemotherapy, Monocyte, Smoking history, TG, Mode of operation, AGR, Adjuvant immunotherapy, Family history of cancer, HCT, PLR, CRP, Gender, Drinking history, LDH, NLR, HGB, Stage, TP, CHO, PLT, MCH, WBC, T
KNN	1 - AUC loss after permutation < 0.26	AGR, Adjuvant chemotherapy, Adjuvant immunotherapy, ALB, ApoA-I, CHO, CRP, Diabetes, Drinking history, Family history of cancer, GLOB, HCT, HDL-C, HGB, Hypertension, LCR, LDL-C, LMR, Lymphocyte, M, MCV, Mode of operation, Monocyte, Neutrophil, NLR, Pathological type, PLR, PLT, Radiotherapy, SII, Smoking history, Stage, TG

Note: SVM = support vector machine, ANN = artificial neural network, KNN = k-nearest neighbor, AUC = area under the curve.

Supplementary Table S2. Baseline characteristics of the study population

Characteristics	All patients (n = 8007)		p value
	ICU cohort (n = 6799)	Non-ICU cohort (n = 1208)	
**Age (years)**			0.0584
Median (range)	64 (19–84)	57 (22–92)	
< 60	2535 (37.28)	485 (40.15)	
≥ 60	4264 (62.72)	723 (59.85)	
**Gender**			0.0853
Male	3957 (58.20)	671 (55.55)	
Female	2842 (41.80)	537 (44.45)	
**Smoking status**			0.1282
Non–smoker	4029 (59.26)	744 (61.59)	
Smoker	2770 (40.74)	464 (38.41)	
**Drinking status**			
Yes	1400 (20.59)	254 (21.03)	0.7306
No	5399 (79.41)	954 (78.97)	
**Hypertension**			
Yes	1320 (19.41)	208 (17.22)	0.0735
No	5479 (80.59)	1000 (82.78)	
**Diabetes**			0.0904
Yes	699 (10.28)	105 (8.69)	
No	6100 (89.72)	1103 (91.31)	
**Family history**			0.1747
Yes	1490 (21.91)	286 (23.68)	
No	5309 (78.09)	922 (76.32)	
**Pathological type**			0.7211
Adenocarcinoma	5187 (76.52)	930 (76.99)	
Squamous carcinoma	1130 (16.67)	200 (16.56)	
Others	482 (6.81)	78 (6.45)	
**Surgical method**			0.2213
Wedge resection	2418 (35.56)	440 (36.42)	
Lobectomy	3007 (44.17)	550 (44.53)	
Pneumonectomy	1374 (20.27)	218 (18.05)	
**T–stage**			0.9256
T1	3703 (54.46)	665 (55.05)	
T2	2173 (31.96)	387 (32.04)	
T3	635 (9.34)	109 (9.02)	
T4	288 (4.24)	47 (3.89)	
**N–stage**			0.9906
N0	4479 (65.88)	790 (65.40)	
N1	1106 (16.27)	200 (16.56)	
N2	1169 (17.19)	210 (17.38)	
N3	45 (0.66)	8 (0.66)	
**M–stage**			0.8483
M0	6675 (98.18)	1185 (98.10)	
M1	124 (1.82)	23 (1.90)	
**Disease stage**			0.0631

I	3779 (55.58)	630 (52.15)	
II	1484 (21.83)	265 (21.94)	
III	1412 (20.77)	290 (24.01)	
IV	124 (1.82)	23 (1.9)	
**Radiotherapy**			0.9619
Yes	402 (5.91)	71 (5.88)	
No	6397 (94.09)	1137 (94.12)	
**Adjuvant Immunotherapy**			0.2153
Yes	274 (4.03)	58 (4.80)	
No	6525 (95.97)	1150 (95.20)	
**Adjuvant chemotherapy**			0.0541
Yes	1366 (20.09)	272 (22.52)	
No	5433 (79.91)	936 (77.48)	

Note: ICU = intensive care unit

Supplementary Table S3. The impact of clinical characteristics on DFS and OS in patients with and without ICU admission

Characteristics	DFS (months), median and range		HR#	*p* value	OS (months), median and range		HR#	*p* value
	Non-ICU cohort	ICU cohort			Non-ICU cohort	ICU cohort		
**Age (years)**								
＜60	NA (0.03–126.07)	NA (0.03–127.33)	1.14	0.4050	NA (0.42–130.3)	NA (0.03–126.87)	0.76	**0.0099**
≥ 60	NA (0.08–128.63)	NA (0.08–126.23)	0.82	**<0.0001**	102.57 (0.08–128.55)	121.13 (0.03–126.23)	0.78	**0.0008**
**Sex**								
Male	NA (0.10–126.53)	NA (0.03–127.33)	1.05	0.1572	95.17 (0.10–130.3)	121.13 (0.03–126.23)	0.81	**0.0054**
Female	NA (0.03–128.63)	NA (0.13–127)	0.75	**<0.0001**	NA (0.08–128.55)	NA (0.1–126.87)	0.66	**0.0002**
**Smoking status**								
Non-smoker	NA (0.03–128.63)	NA (0.03–127)	0.84	**0.0002**	NA (0.08–128.55)	NA (0.03–126.03)	0.68	**<0.0001**
Smoker	NA (0.03–128.63)	NA (0.27–126.53)	1.04	0.1039	92.73 (0.37–130.3)	121.13(0.03–126.87)	0.84	**0.0450**
**Drinking status**								
Yes	NA (0.10–126.07)	NA (0.07–127.33)	1.48	0.6172	95.17 (0.10–126.07)	121.13 (0.03–126.23)	0.91	0.4366
No	NA (0.03–128.63)	NA (0.03–127)	0.81	**<0.0001**	NA (0.08–130.3)	NA (0.03–126.87)	0.72	**<0.0001**
**Hypertension**								
Yes	NA (0.10–121.8)	79.02 (0.07–92.65)	1.04	0.0800	NA (0.10–121.8)	NA (0.03–92.65)	0.87	0.4354
No	NA (0.03–128.63)	NA (0.03–127.33)	0.91	**0.0013**	116.4 (0.08–130.3)	17.73 (0.03–126.87)	0.76	**<0.0001**
**Diabetes**								
Yes	NA (0.47–121.03)	NA (0.1–127.33)	1.93	0.8327	NA (0.47–121.03)	111 (0.1–119.03)	1.46	0.1415
No	NA (0.03–128.63)	NA (0.03–127)	0.86	**0.0001**	116.4 (0.08–130.3)	NA (0.03–126.87)	0.73	**<0.0001**
**Family history**								
Yes	NA (0.3–126.53)	NA (0.1–125.77)	1.20	0.3677	NA (0.47–126.53)	NA (0.03–126.03)	0.92	0.5764
No	NA (0.03–128.63)	NA (0.03–127.33)	0.87	**0.0001**	102.57 (0.08–130.3)	NA (0.03–126.87)	0.74	**<0.0001**
**Pathological type**								
Adenocarcinoma	NA (0.03–128.63)	NA (0.03–127)	0.87	**0.0003**	NA (0.08–128.55)	NA (0.03–126.03)	0.69	**<0.0001**
Squamous	NA (1.03–128.63)	NA (0.43–127.33)	1.12	0.1958	NA (0.8–130.3)	NA (0.03–126.23)	0.97	0.7936
Others	NA (0.47–126.07)	NA (0.47–126.87)	1.68	0.8658	93.6 (0.47–126.07)	111 (0.03–126.87)	0.91	0.6538
**Surgical method**								
Wedge resection	NA (0.03–123.67)	NA (0.03–124.37)	1.01	0.0560	NA (0.10–125.57)	NA (0.07–126.03)	0.71	**0.0069**
Lobectomy	NA (0.53–128.63)	NA (0.13–126.87)	0.85	0.9736	116.4 (0.58–126.53)	121.13 (0.03–126.87)	0.72	0.6205
Pneumonectomy	NA (0.08–126.07)	NA (0.1–127.33)	1.42	**0.0002**	93.83 (0.08–130.3)	NA (0.03–126.23)	1.09	**<0.0001**
**T-stage**								
T1	NA (0.08–126.07)	NA (0.03–124.37)	0.87	**0.001**	NA (0.08–130.3)	NA (0.03–126.03)	0.66	**0.0001**
T2	NA (0.03–128.63)	NA (0.08–127.33)	1.07	0.1559	102.4 (0.37–128.55)	114.13 (0.03–126.23)	0.84	0.0749
T3	116.93(0.10–125.27)	NA (0.08–124.4)	1.06	0.0888	59.1 (0.10–125.27)	87.23 (0.08–124.4)	0.75	0.0541
T4	NA (3.03–121.8)	NA (0.27–126.87)	1.42	0.4350	56.13 (1.17–126.3)	NA (0.23–126.87)	0.98	0.9300
**N-stage**								
N0	NA (0.03–126.53)	NA (0.03–123.37)	0.84	**0.0003**	NA (0.08–130.3)	NA (0.03–126.03)	0.65	**<0.0001**
N1	NA (0.27–123.67)	NA (0.07–127.33)	1.19	0.3701	102.4 (0.42–123.67)	114.13 (0.03–126.03)	0.88	0.3446

N2	116.93 (0.10–128.63)	NA (0.87–127)	1.13	0.2939	55.47 (0.10–126.3)	71.17 (0.08–126.87)	0.85	0.1292
N3	27.13 (8.2–97.03)	80.8 (2.97–116.33)	1.50	0.2355	NA (11.23–97.03)	80.03 (1.37–116.33)	1.30	0.6739
**M-stage**								
M0	NA (0.03–128.63)	NA (0.03–127.33)	0.89	**0.0003**	116.4 (0.08–130.3)	NA (0.03–126.87)	0.76	**<0.0001**
M1	NA (1.77–67.7)	NA (0.93–67.67)	1.52	0.3268	NA (5.97–67.7)	59.7 (0.73–67.67)	1.54	0.3132
**Disease stage**								
I	NA (0.03–126.53)	NA (0.03–122)	0.87	**0.0023**	NA (0.08–130.3)	NA (0.03–126.03)	0.60	**<0.0001**
II	NA (0.27–124.07)	NA (0.07–127.33)	1.14	0.2957	116.4 (0.42–124.07)	NA (0.03–125.77)	0.89	0.3206
III	NA (0.10–128.63)	NA (0.08–127)	1.13	0.3589	56.13 (0.10–126.3)	73.53 (0.08–126.87)	0.59	0.2102
IV	NA (1.77–67.7)	NA (0.93–67.67)	1.52	0.3268	NA (5.97–67.7)	59.7 (0.73–67.67)	1.54	0.3132
**Radiotherapy**								
Yes	60.01 (1.3–121.8)	60.22 (1.73–127)	1.50	0.8815	75.53 (1.3–121.8)	NA (0.23–124.4)	0.97	0.8983
No	NA (0.03–128.63)	NA (0.03–127.33)	0.85	**<0.0001**	118.97 (0.08–130.3)	NA (0.03–126.87)	0.75	**<0.0001**
**Adjuvant chemotherapy**								
Yes	NA (0.08–120.07)	NA (0.08–127.33)	1.57	0.5610	NA (0.08–128.55)	NA (0.03–126.23)	1.02	0.8740
No	NA (0.03–128.63)	NA (0.03–127)	0.82	**<0.0001**	116.4 (0.10–130.3)	NA (0.03–126.87)	0.71	**<0.0001**
**Adjuvant immunotherapy**								
Yes	97.07 (1.3–125.27)	NA (0.3–124.4)	1.84	0.5753	62.7 (1.17–125.57)	71.77 (0.53–126.03)	0.80	0.2453
No	NA (0.03–128.63)	NA (0.03–127.33)	0.86	**0.0001**	NA (0.08–130.3)	NA (0.03–126.87)	0.78	**0.0001**

Note: ICU = intensive care unit, DFS = disease-free survival, OS = overall survival. # ICU cohort vs. non-ICU cohort.

Supplementary Table S4. The alterations of hematologic parameters in all patients

Hematologic parameters	All patients	
	Mean ± SD	*p* value[Φ]
**RBC (×10^{12}/L)**		**< 0.0001**
Level at pre-surgery	5.07 ± 0.02	
Level at post-surgery	4.78 ± 0.01	
Difference[ɩ]	-0.29 ± 0.02	
**WBC (×10^{9}/L)**		**< 0.0001**
Level at pre-surgery	6.64 ± 0.07	
Level at post-surgery	8.67 ± 0.07	
Difference[ɩ]	2.04 ± 0.10	
**Neutrophil (×10^{9}/L)**		0.0645
Level at pre-surgery	4.13 ± 0.07	
Level at post-surgery	8.84 ± 2.54	
Difference[ɩ]	4.71 ± 2.55	
**PLT (×10^{9}/L)**		**< 0.0001**
Level at pre-surgery	222.2 ± 1.54	
Level at post-surgery	248.8 ± 1.44	
Difference[ɩ]	26.63 ± 2.11	
**Lymphocyte (×10^{9}/L)**		**< 0.0001**
Level at pre-surgery	40.17 ± 1.10	
Level at post-surgery	1.71 ± 0.04	
Difference[ɩ]	-38.45 ± 1.10	
**CRP (mg/L)**		**< 0.0001**
Level at pre-surgery	7.63 ± 0.27	
Level at post-surgery	36.77 ± 0.62	
Difference[ɩ]	29.13 ± 0.68	
**Monocyte (×10^{9}/L)**		**< 0.0001**
Level at pre-surgery	21.20 ± 0.57	
Level at post-surgery	0.59 ± 0.01	
Difference[ɩ]	-20.61 ± 0.57	
**SII**		**0.0475**
Level at pre-surgery	606 ± 10.87	
Level at post-surgery	2102 ± 754.7	
Difference[ɩ]	1496 ± 754.8	
**NLR**		0.0900
Level at pre-surgery	2.69 ± 0.05	
Level at post-surgery	10.00 ± 4.31	
Difference[ɩ]	7.310 ± 4.31	

**PLR**		**< 0.0001**
Level at pre-surgery	129.7 ± 1.08	
Level at post-surgery	185.1 ± 1.54	
Difference[l]	55.44 ± 1.88	
**LCR**		**< 0.0001**
Level at pre-surgery	25.51 ± 0.74	
Level at post-surgery	1.325 ± 0.07	
Difference[l]	-24.19 ± 0.74	
**LMR**		**< 0.0001**
Level at pre-surgery	120.4 ± 10.64	
Level at post-surgery	3.74 ± 0.09	
Difference[l]	-116.7 ± 10.64	
**HGB (g/L)**		**< 0.0001**
Level at pre-surgery	124.7 ± 0.56	
Level at post-surgery	130.6 ± 0.44	
Difference[l]	5.91 ± 0.71	
**HCT (%)**		**< 0.0001**
Level at pre-surgery	49.73 ± 0.23	
Level at post-surgery	40.90 ± 0.62	
Difference[l]	-8.83 ± 0.66	
**MCV (fL)**		**< 0.0001**
Level at pre-surgery	82.40 ± 1.17	
Level at post-surgery	90.72 ± 0.14	
Difference[l]	8.32 ± 1.18	
**MCH (pg)**		**< 0.0001**
Level at pre-surgery	36.04 ± 0.18	
Level at post-surgery	29.57 ± 0.03	
Difference[l]	-6.47 ± 0.18	
**ALB (g/L)**		**< 0.0001**
Level at pre-surgery	38.34 ± 0.20	
Level at post-surgery	40.83 ± 0.14	
Difference[l]	2.50 ± 0.25	
**GLOB (g/L)**		**< 0.0001**
Level at pre-surgery	26.50 ± 0.61	
Level at post-surgery	30.77 ± 0.80	
Difference[l]	4.27 ± 1.01	
**AGR**		**< 0.0001**
Level at pre-surgery	28.84 ± 0.79	
Level at post-surgery	1.62 ± 0.05	

Difference[ι]	-27.23 ± 0.79	
**TP (g/L)**		**0.0012**
Level at pre-surgery	67.04 ± 0.44	
Level at post-surgery	71.17 ± 1.20	
Difference[ι]	4.13 ± 1.28	
**CHO (mmol/L)**		0.0578
Level at pre-surgery	4.58 ± 0.07	
Level at post-surgery	4.77 ± 0.05	
Difference[ι]	0.19 ± 0.09	
**HDL–C (mmol/L)**		**< 0.0001**
Level at pre-surgery	1.67 ± 0.03	
Level at post-surgery	1.33 ± 0.03	
Difference[ι]	-0.34 ± 0.04	
**LDL–C (mmol/L)**		**0.0478**
Level at pre-surgery	3.03 ± 0.06	
Level at post-surgery	3.23 ± 0.08	
Difference[ι]	-0.01 ± 0.10	
**TG (mmol/L)**		**0.0010**
Level at pre-surgery	1.48 ± 0.02	
Level at post-surgery	1.63 ± 0.04	
Difference[ι]	-0.15 ± 0.05	
**ApoA–I (g/L)**		**0.0013**
Level at pre-surgery	1.39 ± 0.03	
Level at post-surgery	1.29 ± 0.003	
Difference[ι]	-0.10 ± 0.03	
**Apo–B (g/L)**		**< 0.0001**
Level at pre-surgery	1.31 ± 0.05	
Level at post-surgery	0.97 ± 0.02	
Difference[ι]	-0.34 ± 0.05	
**LDH (U/L)**		**< 0.0001**
Level at pre-surgery	160.3 ± 0.84	
Level at post-surgery	188.9 ± 1.11	
Difference[ι]	28.65 ± 1.38	

Note: Data are presented as mean ± standard deviation. [ι]Difference = post-surgery hematologic parameters – pre-surgery hematologic parameters. [Φ]Compared using the paired t-test.

Supplementary Table S5. The cut-off values of hematologic parameters in all patients

Hematologic parameters	Cut-off value
RBC (×10^{12}/L)	5.5
WBC (×10^9/L)	8.0
Neutrophil (×10^9/L)	5.2
PLT (×10^9/L)	350
Lymphocytes (×10^9/L)	1.2
CRP (mg/L)	5.0
Monocyte (×10^9/L)	0.6
SII	888.0
NLR	2.8
PLR	199
LCR	0.3
LMR	3.0
HGB (g/L)	124.3
HCT (%)	38
MCV (fL)	5.0
MCH (pg)	28.1
ALB (g/L)	41.7
GLOB (g/L)	33.1
AGR	1.3
TP (g/L)	69.7
CHO (mmol/L)	4.9
HDL-C (mmol/L)	1.0
LDL-C (mmol/L)	3.5
TG (mmol/L)	1.5
ApoA-I (g/L)	1.2
Apo-B (g/L)	0.8
LDH (U/L)	180.1

Supplementary Table S6. The impact of dynamic changes in hematologic parameters on DFS and OS in patients with and without ICU admission

Hematologic parameters	DFS (months), median and range		HR#	*p* value	OS (months), median and range		HR#	*p* value
	Non-ICU cohort	ICU cohort			Non-ICU cohort	ICU cohort		
**RBC***								
Elevated	NA (0.08–128.63)	NA (0.03–126.87)	0.78	0.4186	100.53 (0.08–130.3)	NA (0.1–126.87)	0.85	0.0991
Reduced	NA (0.03–126.53)	NA (0.07–127.33)	0.75	0.3829	NA (0.10–128.55)	NA (0.03–126.03)	0.71	0.9230
**WBC***								
Elevated	NA (0.80–128.63)	NA (0.03–126.87)	0.84	0.2627	102.57 (0.08–130.3)	NA (0.07–126.87)	0.81	**0.0099**
Reduced	NA (0.03–126.53)	NA (0.08–	0.65	0.5636	NA (0.0966–128.55)	NA (0.03–126.03)	0.71	0.9495
**Neutrophil***								
Elevated	NA (0.08–128.63)	NA (0.03–126.87)	0.85	0.0744	102.57 (0.08–130.3)	NA (0.03–126.87)	0.81	0.0800
Reduced	NA (0.03–126.53)	NA (0.08–127.33)	0.65	0.4730	NA (0.10–128.55)	NA (0.03–126.03)	0.71	0.4044
**PLT***								
Elevated	NA (0.53–123.97)	NA (0.13–126.87)	0.92	**0.0148**	88.03 (3.67–123.97)	NA (0.13–126.87)	0.82	0.1071
Reduced	NA (0.03–128.63)	NA (0.03–127.33)	0.70	**0.0022**	118.97 (0.08–130.3)	NA (0.03–126.23)	0.75	**0.0001**
**Lymphocyte***								
Elevated	NA (0.03–128.63)	NA (0.03–127.33)	0.69	**<0.0001**	118.97 (0.08–130.3)	NA (0.03–126.87)	0.76	**<0.0001**
Reduced	NA (0.72–126.07)	NA (0.11–123.37)	0.80	0.8064	95.17 (0.37–126.07)	NA (0.1–126.03)	0.77	0.3840
**CRP***								
Elevated	NA (0.03–128.63)	NA (0.03–126.87)	0.74	**0.0003**	116.4 (0.72–125.57)	121.1 (0.03–	0.76	**0.0076**
Reduced	NA (0.08–126.53)	NA (0.07–127.33)	0.82	**0.0336**	118.97 (0.08–130.3)	NA (0.03–126.23)	0.79	**0.0009**
**Monocyte***								
Elevated	NA (0.53–125.27)	NA (0.07–125.37)	0.73	0.4159	NA (0.37–125.27)	NA (0.03–126.03)	0.81	0.2999
Reduced	NA (0.03–128.63)	NA (0.03–127.33)	0.82	**0.0001**	116.4 (0.08–130.3)	NA (0.03–126.87)	0.70	**<0.0001**
**SII***								
Elevated	116.4 (0.03–128.63)	NA (0.10–126.87)	0.77	**0.0183**	NA (0.73–121.13)	NA (0.03–126.87)	0.79	0.1150
Reduced	NA (0.08–126.53)	NA (0.03–127.33)	0.75	**0.0028**	NA (0.08–130.3)	NA (0.03–126.23)	0.73	**<0.0001**
**NLR***								
Elevated	NA (0.03–128.63)	NA (0.10–127)	0.76	**0.0008**	95.43 (0.73–130.3)	100.3 (0.03–	0.73	0.2122
Reduced	NA (0.08–126.53)	NA (0.03–127.33)	0.77	**0.0257**	119.0 (0.08–128.55)	NA (0.03–126.23)	0.83	**<0.0001**
**PLR***								
Elevated	NA (0.03–118.67)	NA (0.10–127.33)	0.86	**0.0040**	NA (0.67–121.13)	111 (0.03–126.87)	0.82	**0.0206**
Reduced	NA (0.08–128.63)	NA (0.03–127)	0.64	**0.0045**	116.4 (0.08–130.3)	NA (0.03–136.23)	0.70	**0.0003**
**LCR***								
Elevated	NA (0.08–126.53)	NA (0.07–127.33)	0.81	**0.0063**	118.97 (0.08–130.3)	NA (0.03–126.23)	0.78	**0.0002**
Reduced	NA (0.03–128.63)	NA (0.03–126.87)	0.75	**0.0031**	116.4 (1.95–121.13)	121.1 (0.03–	0.76	**0.0410**
**LMR***								
Elevated	NA (0.08–126.53)	NA (0.07–127.33)	0.79	**0.0072**	118.97 (0.08–130.3)	NA (0.03–126.23)	0.86	**0.0011**
Reduced	NA (0.03–128.63)	NA (0.03–126.87)	0.75	**0.0014**	95.17 (0.03–126.87)	94.26 (0.53–	0.74	**0.0024**
**HGB***								
Elevated	NA (0.03–128.63)	NA (0.03–126.87)	0.72	**0.0068**	116.4 (0.08–130.3)	NA (0.03–126.87)	0.76	**0.0034**
Reduced	116.9 (0.25–126.07)	NA (0.1–127.33)	0.77	**0.0079**	NA (0.25–126.07)	NA (0.03–126.23)	0.77	**0.0011**

**HCT***								
Elevated	NA (0.03–128.63)	NA (0.03–127.33)	0.71	**0.0027**	116.4 (0.08–128.55)	NA (0.03–126.03)	0.70	**0.0005**
Reduced	NA (0.4–125.27)	NA (0.43–126.87)	0.78	**0.0107**	105.6 (1.95–130.3)	NA (0.18–126.87)	0.79	**0.0062**
**MCV***								
Elevated	NA (0.67–126.53)	NA (0.27–126.87)	0.77	0.5092	85.2 (0.67–126.53)	NA (0.27–126.87)	0.83	0.1711
Reduced	NA (0.03–128.63)	NA (0.03–127.33)	0.75	**0.0002**	116.4 (0.08–130.3)	NA (0.03–126.23)	0.70	**<0.0001**
**MCH***								
Elevated	NA (0.03–128.63)	NA (0.07–127.33)	0.72	**0.0003**	116.4 (0.08–130.3)	NA (0.03–126.87)	0.86	**<0.0001**
Reduced	NA (0.25–123.67)	NA (0.03–124.4)	0.80	0.3549	NA (0.25–123.67)	NA (0.1–124.4)	0.70	0.4789
**ALB***								
Elevated	NA (0.08–128.63)	NA (0.03–127.33)	0.80	**0.0010**	116.4 (0.08–128.55)	NA (0.03–126.03)	0.75	**0.0115**
Reduced	NA (0.03–126.07)	NA (0.13–127)	0.75	0.0698	NA (0.82–130.3)	NA (0.03–126.87)	0.78	**0.0003**
**GLOB***								
Elevated	NA (0.25–125.27)	NA (0.07–123.37)	0.69	0.5830	91.1 (0.25–125.27)	NA (0.07–126.03)	0.81	**0.0339**
Reduced	NA (0.03–128.63)	NA (0.03–127.33)	0.83	**0.0002**	118.97 (0.08–130.3)	NA (0.03–126.87)	0.74	**0.0002**
**AGR***								
Elevated	NA (0.08–128.63)	NA (0.03–127.33)	0.94	**0.0010**	116.4 (0.08–130.3)	NA (0.03–126.87)	0.67	**0.0009**
Reduced	NA (0.25–125.27)	NA (0.11–121.93)	0.70	0.0894	95.43 (0.25–125.27)	NA (0.11–121.93)	0.83	**0.0019**
**TP***								
Elevated	NA (0.08–128.63)	NA (0.03–127.33)	0.78	**0.0084**	119.0 (0.08–128.55)	NA (0.03–126.03)	0.78	**0.0014**
Reduced	NA (0.03–126.53)	NA (0.13–127)	0.75	**0.0074**	116.4 (0.8–130.3)	NA (0.03–126.87)	0.77	**0.0052**
**CHO***								
Elevated	NA (0.08–128.63)	NA (0.03–125.77)	0.72	0.0689	119.0 (0.08–128.55)	121.1 (0.03–	0.81	**0.0341**
Reduced	NA (0.03–126.07)	NA (0.07–127.33)	0.79	**0.0008**	105.6 (0.10–130.3)	NA (0.07–126.87)	0.75	**0.0001**
**HDL-C***								
Elevated	NA (0.03–128.63)	NA (0.03–127.33)	0.64	**0.0022**	116.4 (0.10–130.3)	NA (0.03–126.87)	0.82	**<0.0001**
Reduced	NA (0.08–123.97)	NA (0.18–121.1)	0.84	**0.0232**	NA (0.08–123.97)	NA (0.03–120.97)	0.74	0.1647
**LDL-C***								
Elevated	NA (1.07–47.4)	NA (1.03–23.53)	0.71	0.2530	NA (0.25–126.53)	121.1 (0.03–	0.85	0.1408
Reduced	NA (0.03–126.07)	NA (0.03–127.33)	0.78	**0.0002**	105.6 (0.08–130.3)	NA (0.03–126.87)	0.73	**<0.0001**
**TG***								
Elevated	NA (0.03–128.63)	NA (0.03–127.33)	0.76	**0.0002**	104.7 (0.08–130.3)	NA (0.03–126.03)	0.81	**<0.0001**
Reduced	NA (0.10–124.07)	NA (0.07–126.87)	0.77	0.2078	119.0 (0.10–124.07)	NA (0.03–126.87)	0.73	0.1845
**ApoA-I***								
Elevated	NA (0.10–126.53)	NA (0.03–127.33)	0.63	**0.0280**	116.4 (0.10–130.3)	NA (0.03–126.23)	0.87	**0.0046**
Reduced	NA (0.03–128.63)	NA (0.1–127)	0.84	**0.0010**	NA (0.08–126.07)	NA (0.03–126.87)	0.72	**0.0009**
**Apo-B***								
Elevated	NA (0.03–128.63)	NA (0.03–127.33)	0.75	**0.0001**	116.4 (0.08–130.3)	NA (0.03–126.23)	0.92	**<0.0001**
Reduced	NA (0.47–123.67)	NA (0.13–126.87)	0.76	0.7786	102.6 (0.47–123.67)	NA 0.13–126.87)	0.70	0.4659
**LDH***								
Elevated	NA (0.08–128.63)	NA (0.03–127.33)	0.65	**0.0219**	NA (0.08–128.55)	NA (0.03–126.03)	0.72	0.1976
Reduced	NA (0.03–126.53)	NA (0.08–127)	0.94	**0.0043**	116.4 (0.10–130.3)	NA (0.03–126.87)	0.83	**<0.0001**

Note: ICU = intensive care unit, DFS = disease-free survival, OS = overall survival. *Comparison between hematologic indications at post-surgery and hematologic indications at pre-surgery. # ICU cohort vs. non-ICU cohort.

Supplementary Table S7. The impact of baseline hematologic parameters on DFS and OS in patients with and without ICU admission

Hematologic parameters	DFS (months), median and range		HR[#]	*p* value	OS (months), median and range		HR[#]	*p* value
	Non–ICU cohort	ICU cohort			Non–ICU cohort	ICU cohort		
**RBC**								
High	NA (0.08–128.63)	NA (0.03–126.87)	0.91	0.4186	100.53 (0.08–130.3)	NA (0.1–126.87)	0.84	0.0991
Low	NA (0.03–126.53)	NA (0.07–127.33)	0.89	0.3829	NA (0.10–128.55)	NA (0.03–126.03)	1.01	0.9230
**WBC**								
High	NA (0.80–128.63)	NA (0.03–126.87)	0.89	0.2627	102.57 (0.08–130.3)	NA (0.07–126.87)	0.78	**0.0099**
Low	NA (0.03–126.53)	NA (0.08–127.33)	0.92	0.5636	NA (0.0966–128.55)	NA (0.03–126.03)	1.01	0.9495
**Neutrophil**								
High	NA (0.08–128.63)	NA (0.03–126.87)	0.81	0.0744	102.57 (0.08–130.3)	NA (0.03–126.87)	0.84	0.0800
Low	NA (0.03–126.53)	NA (0.08–127.33)	0.91	0.4730	NA (0.10–128.55)	NA (0.03–126.03)	0.92	0.4044
**PLT**								
High	NA (0.53–123.97)	NA (0.13–126.87)	0.58	**0.0148**	88.03 (3.67–123.97)	NA (0.13–126.87)	0.74	0.1071
Low	NA (0.03–128.63)	NA (0.03–127.33)	0.79	**0.0022**	118.97 (0.08–130.3)	NA (0.03–126.23)	0.77	**0.0001**
**Lymphocyte**								
High	NA (0.03–128.63)	NA (0.03–127.33)	0.74	**<0.0001**	118.97 (0.08–130.3)	NA (0.03–126.87)	0.76	**< 0.0001**
Low	NA (0.72–126.07)	NA (0.11–123.37)	1.08	0.8064	95.17 (0.37–126.07)	NA (0.1–126.03)	0.83	0.3840
**CRP**								
High	NA (0.03–128.63)	NA (0.03–126.87)	0.61	**0.0003**	116.4 (0.72–125.57)	121.1 (0.03–126.87)	0.73	**0.0076**
Low	NA (0.08–126.53)	NA (0.07–127.33)	0.83	**0.0336**	118.97 (0.08–130.3)	NA (0.03–126.23)	0.78	**0.0009**
**Monocyte**								
High	NA (0.53–125.27)	NA (0.07–125.37)	0.88	0.4159	NA (0.37–125.27)	NA (0.03–126.03)	0.88	0.2999
Low	NA (0.03–128.63)	NA (0.03–127.33)	0.72	**0.0001**	116.4 (0.08–130.3)	NA (0.03–126.87)	0.74	**< 0.0001**
**SII**								
High	116.4 (0.03–128.63)	NA (0.10–126.87)	0.67	**0.0183**	NA (0.73–121.13)	NA (0.03–126.87)	0.80	0.1150
Low	NA (0.08–126.53)	NA (0.03–127.33)	0.78	**0.0028**	NA (0.08–130.3)	NA (0.03–126.23)	0.76	**< 0.0001**
**NLR**								
High	NA (0.03–128.63)	NA (0.10–127)	0.64	**0.0008**	95.43 (0.73–130.3)	100.3 (0.03–126.87)	0.87	0.2122
Low	NA (0.08–126.53)	NA (0.03–127.33)	0.82	**0.0257**	119.0 (0.08–128.55)	NA (0.03–126.23)	0.73	**< 0.0001**
**PLR**								
High	NA (0.03–118.67)	NA (0.10–127.33)	0.59	**0.0040**	NA (0.67–121.13)	111 (0.03–126.87)	0.70	**0.0206**
Low	NA (0.08–128.63)	NA (0.03–127)	0.80	**0.0045**	116.4 (0.08–130.3)	NA (0.03–136.23)	0.78	**0.0003**
**LCR**								
High	NA (0.08–126.53)	NA (0.07–127.33)	0.80	**0.0063**	118.97 (0.08–130.3)	NA (0.03–126.23)	0.77	**0.0002**
Low	NA (0.03–128.63)	NA (0.03–126.87)	0.60	**0.0031**	116.4 (1.95–121.13)	121.1 (0.03–126.87)	0.75	**0.0410**
**LMR**								
High	NA (0.08–126.53)	NA (0.07–127.33)	0.80	**0.0072**	118.97 (0.08–130.3)	NA (0.03–126.23)	0.80	**0.0011**
Low	NA (0.03–128.63)	NA (0.03–126.87)	0.57	**0.0014**	95.17 (0.03–126.87)	94.26 (0.53–126.3)	0.64	**0.0024**
**HGB**								
High	NA (0.03–128.63)	NA (0.03–126.87)	0.79	**0.0068**	116.4 (0.08–130.3)	NA (0.03–126.87)	0.80	**0.0034**
Low	116.9 (0.25–126.07)	NA (0.1–127.33)	0.70	**0.0079**	NA (0.25–126.07)	NA (0.03–126.23)	0.69	**0.0011**

**HCT**								
High	NA (0.03–128.63)	NA (0.03–127.33)	0.79	**0.0027**	116.4 (0.08–128.55)	NA (0.03–126.03)	0.79	**0.0005**
Low	NA (0.4–125.27)	NA (0.43–126.87)	0.61	**0.0107**	105.6 (1.95–130.3)	NA (0.18–126.87)	0.63	**0.0062**
**MCV**								
High	NA (0.67–126.53)	NA (0.27–126.87)	0.85	0.5092	85.2 (0.67–126.53)	NA (0.27–126.87)	0.77	0.1711
Low	NA (0.03–128.63)	NA (0.03–127.33)	0.75	**0.0002**	116.4 (0.08–130.3)	NA (0.03–126.23)	0.77	**< 0.0001**
**MCH**								
High	NA (0.03–128.63)	NA (0.07–127.33)	0.75	**0.0003**	116.4 (0.08–130.3)	NA (0.03–126.87)	0.75	**< 0.0001**
Low	NA (0.25–123.67)	NA (0.03–124.4)	0.83	0.3549	NA (0.25–123.67)	NA (0.1–124.4)	0.89	0.4789
**ALB**								
High	NA (0.08–128.63)	NA (0.03–127.33)	0.74	**0.0010**	116.4 (0.08–128.55)	NA (0.03–126.03)	0.82	**0.0115**
Low	NA (0.03–126.07)	NA (0.13–127)	0.80	0.0698	NA (0.82–130.3)	NA (0.03–126.87)	0.69	**0.0003**
**GLOB**								
High	NA (0.25–125.27)	NA (0.07–123.37)	0.89	0.5830	91.1 (0.25–125.27)	NA (0.07–126.03)	0.72	**0.0339**
Low	NA (0.03–128.63)	NA (0.03–127.33)	0.74	**0.0002**	118.97 (0.08–130.3)	NA (0.03–126.87)	0.78	**0.0002**
**AGR**								
High	NA (0.08–128.63)	NA (0.03–127.33)	0.77	**0.0010**	116.4 (0.08–130.3)	NA (0.03–126.87)	0.80	**0.0009**
Low	NA (0.25–125.27)	NA (0.11–121.93)	0.72	0.0894	95.43 (0.25–125.27)	NA (0.11–121.93)	0.63	**0.0019**
**TP**								
High	NA (0.08–128.63)	NA (0.03–127.33)	0.94	**0.0084**	119.0 (0.08–128.55)	NA (0.03–126.03)	0.79	**0.0014**
Low	NA (0.03–126.53)	NA (0.13–127)	0.9	**0.0074**	116.4 (0.8–130.3)	NA (0.03–126.87)	0.74	**0.0052**
**CHO**								
High	NA (0.08–128.63)	NA (0.03–125.77)	0.82	0.0689	119.0 (0.08–128.55)	121.1 (0.03–126.03)	0.82	**0.0341**
Low	NA (0.03–126.07)	NA (0.07–127.33)	0.72	**0.0008**	105.6 (0.10–130.3)	NA (0.07–126.87)	0.73	**0.0001**
**HDL-C**								
High	NA (0.03–128.63)	NA (0.03–127.33)	0.78	**0.0022**	116.4 (0.10–130.3)	NA (0.03–126.87)	0.76	**< 0.0001**
Low	NA (0.08–123.97)	NA (0.18–121.1)	0.66	**0.0232**	NA (0.08–123.97)	NA (0.03–120.97)	0.79	0.1647
**LDL-C**								
High	NA (1.07–47.4)	NA (0.03–123.53)	0.85	0.2530	NA (0.25–126.53)	121.1 (0.03–126.03)	0.84	0.1408
Low	NA (0.03–126.07)	NA (0.03–127.33)	0.73	**0.0002**	105.6 (0.08–130.3)	NA (0.03–126.87)	0.74	**< 0.0001**
**TG**								
High	NA (0.03–128.63)	NA (0.03–127.33)	0.72	**0.0002**	104.7 (0.08–130.3)	NA (0.03–126.03)	0.73	**< 0.0001**
Low	NA (0.10–124.07)	NA (0.07–126.87)	0.84	0.2078	119.0 (0.10–124.07)	NA (0.03–126.87)	0.86	0.1845
**ApoA-I**								
High	NA (0.10–126.53)	NA (0.03–127.33)	0.82	**0.0280**	116.4 (0.10–130.3)	NA (0.03–126.23)	0.80	**0.0046**
Low	NA (0.03–128.63)	NA (0.1–127)	0.67	**0.0010**	NA (0.08–126.07)	NA (0.03–126.87)	0.70	**0.0009**
**Apo-B**								
High	NA (0.03–128.63)	NA (0.03–127.33)	0.73	**0.0001**	116.4 (0.08–130.3)	NA (0.03–126.23)	0.75	**< 0.0001**
Low	NA (0.47–123.67)	NA (0.13–126.87)	0.95	0.7786	102.6 (0.47–123.67)	NA (0.13–126.87)	0.89	0.4659
**LDH**								
High	NA (0.08–128.63)	NA (0.03–127.33)	0.75	**0.0219**	NA (0.08–128.55)	NA (0.03–126.03)	0.87	0.1976
Low	NA (0.03–126.53)	NA (0.08–127)	0.77	**0.0043**	116.4 (0.10–130.3)	NA (0.03–126.87)	0.71	**< 0.0001**

Note: ICU = intensive care unit, DFS = disease-free survival, OS = overall survival. # ICU cohort vs. non-ICU cohort.

Supplementary Table S8. The impact of post-surgery hematologic parameters on DFS and OS in patients with and without ICU admission

Hematologic parameters	DFS (months), median and range		HR#	*p* value	OS (months), median and range		HR#	*p* value
	Non-ICU cohort	ICU cohort			Non-ICU cohort	ICU cohort		
**RBC**								
High	NA (0.08–123.67)	NA (0.03–126.23)	0.75	0.0516	95.43 (0.08–130.3)	NA (0.07–126.23)	0.81	0.0565
Low	NA (0.03–128.63)	NA (0.08–127.33)	0.77	**0.0019**	NA (0.10–126.53)	NA (0.03–126.87)	0.78	**0.0007**
**WBC**								
High	NA (0.80–128.63)	NA (0.03–126.33)	0.77	**0.0358**	104.7 (0.08–126.53)	NA (0.07–124.4)	0.82	**0.0374**
Low	NA (0.03–126.07)	NA (0.1–127)	0.78	**0.0051**	NA (0.0966–130.3)	NA (0.03–126.87)	0.72	**< 0.0001**
**Neutrophil**								
High	NA (0.08–128.63)	NA (0.03–126.23)	0.77	**0.0345**	116.4 (0.08–126.53)	NA (0.07–124.4)	0.82	**0.0278**
Low	NA (0.03–126.07)	NA (0.1–127)	0.77	**0.0048**	NA (0.42–130.3)	NA (0.03–126.87)	0.72	**0.0001**
**PLT**								
High	NA (0.53–117。57)	NA (0.18–126.23)	0.77	0.2648	105.6 (0.8–117.57)	NA (0.13–126.23)	0.80	0.2699
Low	NA (0.03–128.63)	NA (0.03–127.33)	0.76	**0.0004**	118.97 (0.08–130.3)	NA (0.03–126.87)	0.76	**< 0.0001**
**Lymphocyte**								
High	NA (0.03–128.63)	NA (0.08–127.33)	0.75	**0.0018**	116.4 (0.25–126.3)	NA (0.03–126.87)	0.76	**0.0002**
Low	NA (0.08–126.53)	NA (0.03–127)	0.77	**0.0458**	105.6 (0.08–130.3)	NA (0.03–126.23)	0.80	**0.0394**
**CRP**								
High	NA (0.03–125.27)	NA (0.08–126.87)	0.70	**0.0004**	104.7 (0.08–125.27)	NA (0.03–126.87)	0.77	**0.0017**
Low	NA (0.25–128.63)	NA (0.03–127.33)	0.84	0.1107	118.97 (0.25–130.3)	NA (0.03–126.23)	0.78	**0.0059**
**Monocyte**								
High	NA (0.08–122.67)	NA (0.1–127.33)	0.76	**0.0267**	NA (0.08–128.55)	NA (0.03–126.87)	0.68	**0.0004**
Low	NA (0.03–128.63)	NA (0.03–127)	0.76	**0.0031**	116.4 (0.25–130.3)	NA (0.03–126.03)	0.81	**0.0071**
**SII**								
High	NA (0.03–126.07)	NA (0.08–126.87)	0.70	**0.0009**	95.43 (0.08–126.3)	NA (0.03–126.87)	0.72	**0.0002**
Low	NA (0.25–128.63)	NA (0.03–127.33)	0.82	0.0511	118.97 (0.25–130.3)	NA (0.03–126.03)	0.82	**0.0256**
**NLR**								
High	NA (0.03–126.07)	NA (0.08–126.87)	0.76	**0.0049**	104.7 (0.08–126.3)	NA (0.03–126.87)	0.76	**0.0009**
Low	NA (0.25–128.63)	NA (0.03–127.33)	0.77	**0.0169**	119.0 (0.25–130.3)	121.1 (0.03–126.03)	0.79	**0.0119**
**PLR**								
High	NA (0.03–126.07)	NA (0.1–126.87)	0.76	**0.0354**	89.73 (0.10–126.3)	NA (0.1–126.87)	0.74	**0.0053**
Low	NA (0.08–128.63)	NA (0.03–127.33)	0.76	**0.0022**	NA (0.08–130.3)	NA (0.03–126.23)	0.78	**0.0013**
**LCR**								
High	NA (0.25–128.63)	NA (0.03–127.33)	0.85	0.1549	118.97 (0.25–130.3)	NA (0.03–126.23)	0.80	**0.0131**
Low	NA (0.03–125.27)	NA (0.08–126.87)	0.69	**0.0002**	104.7 (0.08–125.27)	NA (0.03–126.87)	0.75	**0.0006**
**LMR**								
High	NA (0.25–128.63)	NA (0.03–127.33)	0.78	**0.0179**	118.97 (0.25–130.3)	NA (0.03–126.23)	0.79	**0.0099**
Low	NA (0.03–125.27)	NA (0.08–126.87)	0.75	**0.0047**	104.7 (0.08–125.27)	NA (0.03–126.87)	0.76	**0.0011**
**HGB**								
High	NA (0.03–128.63)	NA (0.03–126.87)	0.73	**0.0009**	116.4 (0.08–126.53)	NA (0.03–126.87)	0.80	**0.0041**
Low	116.9 (0.3–125.27)	NA (0.08–127.33)	0.82	0.0898	NA (0.37–130.3)	NA (0.03–126.23)	0.73	**0.0014**

**HCT**								
High	NA (0.08–126.53)	NA (0.03–127.33)	0.80	**0.0165**	116.4 (0.08–130.3)	NA (0.03–126.87)	0.77	**0.0006**
Low	NA (0.03–128.63)	NA (0.07–122.97)	0.69	**0.0025**	NA (0.37–128.55)	NA (0.07–122.97)	0.77	**0.0111**
**MCV**								
High	NA (0.4–126.07)	NA (0.03–126.87)	0.65	**0.0228**	102.6 (0.53–128.55)	114.13 (0.1–126.87)	0.80	0.1649
Low	NA (0.03–128.63)	NA (0.07–127.33)	0.78	**0.0021**	116.4 (0.08–130.3)	NA (0.03–126.23)	0.76	**< 0.0001**
**MCH**								
High	NA (0.03–128.63)	NA (0.03–127.33)	0.75	**0.0005**	116.4 (0.08–130.3)	NA (0.03–126.03)	0.78	**0.0003**
Low	NA (0.25–126.07)	NA (0.07–126.87)	0.79	0.1980	100.5 (0.25–126.07)	NA (0.07–126.87)	0.73	**0.0277**
**ALB**								
High	NA (0.25–128.63)	NA (0.03–127.33)	0.82	0.0618	119.0 (0.25–130.3)	NA (0.03–126.03)	0.79	**0.0103**
Low	NA (0.03–125.27)	NA (0.08–126.87)	0.71	**0.0009**	100.5 (0.08–125.27)	NA (0.03–126.87)	0.76	**0.0009**
**GLOB**								
High	NA (0.10–116.87)	NA (0.23–126.87)	0.80	0.2115	NA (0.10–126.3)	121.1 (0.03–126.87)	0.83	0.2190
Low	NA (0.03–128.63)	NA (0.03–127.33)	0.75	**0.0005**	116.4 (0.08–130.3)	NA (0.03–126.03)	0.76	**< 0.0001**
**AGR**								
High	NA (0.03–128.63)	NA (0.03–127.33)	0.78	**0.0057**	105.6 (0.37–130.3)	NA (0.03–126.03)	0.73	**< 0.0001**
Low	NA (0.08–125.27)	NA (0.1–126.87)	0.73	**0.0147**	NA (0.08–126.3)	121.1 (0.03–126.87)	0.87	0.2172
**TP**								
High	NA (0.08–128.63)	NA (0.03–127.33)	0.73	**0.0013**	119.0 (0.08–130.3)	NA (0.03–126.87)	0.79	**0.0070**
Low	NA (0.03–125.27)	NA (0.08–127)	0.80	**0.0445**	102.4 (0.37–125.27)	NA (0.03–125.77)	0.75	**0.0015**
**CHO**								
High	NA (0.25–126.53)	NA (0.03–127)	0.71	**0.0036**	119.0 (0.25–126.53)	NA (0.03–126.03)	0.81	**0.0342**
Low	NA (0.03–128.63)	NA (0.08–127.33)	0.79	**0.0167**	104.7 (0.08–130.3)	NA (0.03–126.87)	0.75	**0.0002**
**HDL-C**								
High	NA (0.03–128.63)	NA (0.03–127)	0.76	**0.0013**	119.0 (0.25–130.3)	NA (0.03–126.87)	0.78	**0.0006**
Low	NA (0.08–123.97)	NA (0.1–127.33)	0.75	0.0562	102.6 (0.08–123.97)	NA (0.1–127.33)	0.73	**0.0121**
**LDL-C**								
High	116.9 (0.47–126.07)	NA (0.03–127)	0.58	**<0.0001**	119.0 (0.47–126.3)	NA (0.03–124.4)	0.79	0.0676
Low	NA (0.03–128.63)	NA (0.08–127.33)	0.84	**0.0454**	116.4 (0.08–130.3)	NA (0.03–126.87)	0.76	**0.0001**
**TG**								
High	NA (0.03–126.07)	NA (0.03–127.33)	0.76	**0.0024**	116.4 (0.08–128.55)	NA (0.03–126.23)	0.78	**0.0010**
Low	NA (0.25–128.63)	NA (0.08–126.87)	0.76	**0.0393**	NA (0.25–130.3)	NA (0.03–126.87)	0.75	**0.0086**
**ApoA-I**								
High	NA (0.03–128.63)	NA (0.03–127)	0.78	**0.0078**	116.4 (0.25–130.3)	NA (0.03–126.87)	0.74	**0.0002**
Low	NA (0.08–123.97)	NA (0.08–127.33)	0.74	**0.0093**	NA (0.08–128.55)	NA (0.03–126.23)	0.81	**0.0375**
**Apo-B**								
High	NA (0.03–128.63)	NA (0.03–127.33)	0.69	**<0.0001**	104.7 (0.08–130.3)	NA (0.03–126.87)	0.72	**< 0.0001**
Low	NA (0.10–116.23)	NA (0.08–124.37)	1.00	0.9983	NA (0.10–121.13)	NA (0.03–124.37)	0.89	0.3470
**LDH**								
High	NA (0.03–128.63)	NA (0.07–127.33)	0.74	**0.0051**	102.6 (0.08–126.53)	NA (0.03–126.87)	0.69	**< 0.0001**
Low	NA (0.25–126.07)	NA (0.03–126.23)	0.78	**0.0161**	NA (0.25–130.3)	NA (0.03–126.23)	0.86	0.0801

Note: ICU = intensive care unit, DFS = disease-free survival, OS = overall survival. # ICU cohort vs. non-ICU cohort.

Supplementary Table S9. Summary performance metrics of all four models based on baseline hematologic parameters

Model	Point estimate (95% CI); (95% PI)	
	AUC (Estimated using random effects from bootstrap (R=2000))	Brier score
**Training Cohort**		
SVM	0.88008 (0.87971 – 0.88045); (0.86361 – 0.89655)	0.1296714
CatBoost	0.90526 (0.90491 – 0.90561); (0.88941 – 0.92111)	0.1242914
ANN	0.85717 (0.85673 – 0.85761); (0.83736 – 0.87697)	0.1310779
KNN	0.86059 (0.86016 – 0.86103); (0.84127 – 0.87992)	0.1356239
**Internal Validation Cohort**		
SVM	0.95099 (0.95085 – 0.95114); (0.94455 – 0.95744)	0.1254467
CatBoost	0.97824 (0.97817 – 0.97832); (0.97476 – 0.98173)	0.0932541
ANN	0.88275 (0.88249 – 0.88301); (0.87134 – 0.89415)	0.1257812
KNN	0.93125 (0.93111 – 0.93140); (0.92447 – 0.93804)	0.1181792

Note: ANN = artificial neural network, AUC = area under the curve, KNN = k-nearest neighbors, SVM = support vector machine.
